# α-Tocopherol-13′-Carboxychromanol Induces Cell Cycle Arrest and Cell Death by Inhibiting the SREBP1-SCD1 Axis and Causing Imbalance in Lipid Desaturation

**DOI:** 10.3390/ijms24119229

**Published:** 2023-05-25

**Authors:** Sijia Liao, André Gollowitzer, Lisa Börmel, Charlotte Maier, Luisa Gottschalk, Oliver Werz, Maria Wallert, Andreas Koeberle, Stefan Lorkowski

**Affiliations:** 1 Institute of Nutritional Sciences, Friedrich Schiller University Jena, 07743 Jena, Germany; sijia.liao@uni-jena.de (S.L.); lisa.boermel@uni-jena.de (L.B.); charlottemaier96@gmail.com (C.M.); luisagottschalk@aol.com (L.G.); maria.wallert@uni-jena.de (M.W.); 2 Competence Cluster for Nutrition and Cardiovascular Health (nutriCARD) Halle-Jena-Leipzig, 07743 Jena, Germany; 3 Michael Popp Institute and Center for Molecular Biosciences Innsbruck (CMBI), University of Innsbruck, 6020 Innsbruck, Austria; 4 Department of Pharmaceutical/Medicinal Chemistry, Institute of Pharmacy, Friedrich Schiller University Jena, 07743 Jena, Germany; oliver.werz@uni-jena.de

**Keywords:** α-tocopherol-derived long-chain metabolite, α-T-13′-COOH, apoptosis, adaptive stress response, lipid desaturation, SCD1, SREBP1, macrophages

## Abstract

α-Tocopherol-13′-carboxychromanol (α-T-13′-COOH) is an endogenously formed bioactive α-tocopherol metabolite that limits inflammation and has been proposed to exert lipid metabolism-regulatory, pro-apoptotic, and anti-tumoral properties at micromolar concentrations. The mechanisms underlying these cell stress-associated responses are, however, poorly understood. Here, we show that the induction of G_0_/G_1_ cell cycle arrest and apoptosis in macrophages triggered by α-T-13′-COOH is associated with the suppressed proteolytic activation of the lipid anabolic transcription factor sterol regulatory element-binding protein (SREBP)1 and with decreased cellular levels of stearoyl-CoA desaturase (SCD)1. In turn, the fatty acid composition of neutral lipids and phospholipids shifts from monounsaturated to saturated fatty acids, and the concentration of the stress-preventive, pro-survival lipokine 1,2-dioleoyl-*sn*-glycero-3-phospho-(1′-myo-inositol) [PI(18:1/18:1)] decreases. The selective inhibition of SCD1 mimics the pro-apoptotic and anti-proliferative activity of α-T-13′-COOH, and the provision of the SCD1 product oleic acid (C18:1) prevents α-T-13′-COOH-induced apoptosis. We conclude that micromolar concentrations of α-T-13′-COOH trigger cell death and likely also cell cycle arrest by suppressing the SREBP1-SCD1 axis and depleting cells of monounsaturated fatty acids and PI(18:1/18:1).

## 1. Introduction

The term vitamin E is widely used for a group of fat-soluble organic molecules. Recent studies on vitamin E metabolism postulate that the endogenously formed long-chain metabolites (LCMs) of vitamin E are bioactive molecules with relevance for the regulation of the physiologic functions of vitamin E in humans [1,2,3]. Thereinto, the carboxylic form of the LCMs, which is formed by α-tocopherol (TOH) via cytochrome P450 (CYP450)-dependent ω-hydroxylation and the subsequent ω-oxidation of the side-chain, exerts biological properties, which occur at lower concentrations but show superior and even different effects compared to its precursor [4,5,6].

The endogenous metabolism of vitamin E occurs mainly in the liver [7]. In particular, α-tocopherol-13′-carboxychromanol (α-T-13′-COOH) is present in human serum, and its levels are elevated after oral administration of α-TOH [4,5,8]. This result provides evidence for the systemic relevance of the metabolite in the human body. Moreover, the selective enrichment of α-T-13′-COOH has been observed in human leukocytes, which results in micromolar intracellular concentrations of α-T-13′-COOH and leads to the potent inhibition of the pro-inflammatory enzyme 5-lipoxygenase (5-LO) at sub-micromolar concentrations (IC_50_ = 0.08 µM) [5]. In addition, protection against lipotoxicity by induced expression of the lipid droplet-associated protein perilipin-2 (PLIN2) has also been observed at sub-micromolar concentrations in human macrophages [9]. Other regulatory effects of α-T-13′-COOH have been found for inflammation [5,8,10,11,12,13], cancer [14,15], and foam cell formation [4,6,9], as well as on signaling cascades, including the activation of nuclear receptors [16,17] at micromolar, potentially pharmacologically relevant concentrations in vitro.

Many studies, including preclinical and human trials, have been performed to elucidate the role of vitamin E and its derivatives in cancer (reviewed in [18,19,20]). Recent findings point out that α-T-13′-COOH induces apoptosis in different cancer cell lines, which has not been observed for its precursor [15,21,22]. First of all, the pro-apoptotic potential of α-T-13′-COOH related to mitochondrial damage was demonstrated in HepG2 cells (IC_50_ = 13.5 µM) [15]. Next, the effect of α-T-13′-COOH on cell viability in non-cancer cells was investigated, and no effect was found up to 10 µM in peripheral blood mononuclear cells [5]. Later, Jang et al. reported the anti-proliferative and pro-apoptotic effects of δ-T-13′-COOH (IC_50_ = 8.9 µM) in human colon carcinoma cells, and proposed an interference with sphingolipid metabolism as a possible mechanism [22].

Fatty acid metabolism plays a central role in the regulation of cellular stress responses, among others, impacting proliferation, survival, resistance, stemness, and plasticity [23,24]. The major enzymes and regulatory factors involved in de novo lipogenesis are under the control of sterol regulatory element-binding protein (SREBP)1, which belongs to the family of basic-helix-loop-helix leucine zipper (bHLH-LZ) motif-containing transcription factors. SREBP1 promotes the expression of lipogenic enzymes, such as fatty acid synthase (FAS), acetyl-CoA carboxylase (ACC), and stearoyl-CoA desaturase (SCD)1 [25,26]. Thereinto, SCD1 has emerged as a key player involved in lipid metabolism and tumorigenesis [27]. SCD1 prevents ER stress and apoptosis, reduces the susceptibility to ferroptosis, and controls stress-activated responses, such as the unfolded protein response (UPR), autophagy, and stress-activated protein kinases [28,29,30,31,32]. Numerous lines of evidence suggest that SCD1 activity and MUFA formation are positively associated with resistance to cytotoxic stress and the enhanced survival and metastasis of cancer cells [33]. As an endoplasmic reticulum (ER)-anchored membrane protein, SCD1 catalyzes the insertion of a Δ9 *cis*-double bond into acyl-CoA, preferentially palmitoyl (16:0)-CoA and stearoyl (18:0)-CoA, to produce palmitoleoyl (16:1)-CoA and oleoyl (18:1)-CoA, respectively. SCD1 deficiency causes a substantial reduction in the ratio of monounsaturated fatty acids (MUFA) to saturated fatty acids (SFA) in major membrane phospholipids, storage triglycerides, and free fatty acids [34]. How this metabolic switch is translated into biological function is a matter of debate, and multiple mechanisms have been proposed [28,35,36]. Recently, the phospholipid 1,2-dioleoyl-*sn*-glycero-3-phospho-(1′-myo-inositol) [PI(18:1/18:1)] has been identified as an SCD1-derived signaling lipid that links the activity of SCD1 to cellular stress responses, cell proliferation, and apoptosis [37].

To gain deeper insights into the role of the vitamin E-derived metabolite α-T-13′-COOH in the regulation of cytotoxic stress and the adaptive stress response, we focused on the regulatory effects of α-T-13′-COOH on cell proliferation, cell cycle distribution, programmed cell death, and the modulation of stress-regulatory signaling. Notably, α-T-13′-COOH has been reported to be cytotoxic to several cancer cell lines [4,15], but is well tolerated by primary human peripheral blood mononuclear cells [5]. Given the strong accumulation of α-T-13′-COOH in innate immune cells [5], we speculated about selective cancer cell lethality and focused further studies on the cytotoxic activity of RAW264.7 myeloid leukemia cells. Here, we provide evidence that the endogenously formed α-TOH-derived metabolite α-T-13′-COOH interferes with the proteolytic activation of SREBP1, lowers cellular protein levels of SCD1, and decreases the desaturation of fatty acids in cellular lipids, which in turn causes a decrease in cell-protective PI(18:1/18:1) and triggers apoptosis and G_0_/G_1_-cell cycle arrest.

## 2. Results

### 2.1. α-T-13′-COOH Suppresses Cell Proliferation following G_0_/G_1_ Cell Cycle Arrest

**Figure 1:**
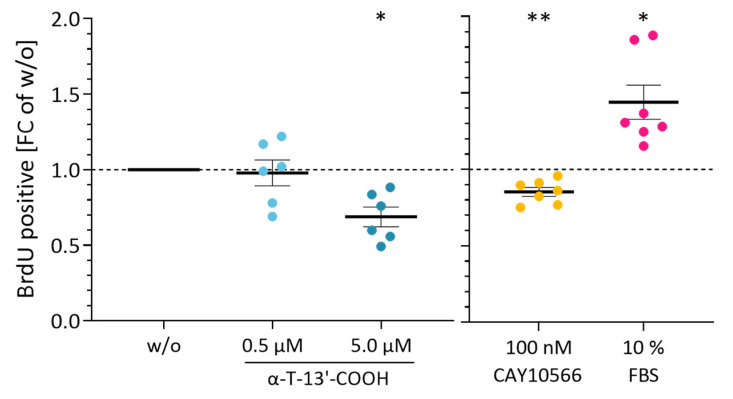
α-T-13′-COOH suppresses proliferation of murine macrophages. The influence of α-T-13′-COOH and the SCD1 inhibitor CAY10566 on the proliferation of murine RAW264.7 cells was analyzed by colorimetric detection of BrdU incorporation into DNA synthesized during cell division. Cells were treated with either vehicle control (DMSO, w/o), α-T-13′-COOH (0.5 µM, 5 µM), the SCD1 inhibitor CAY10566 (100 nM) in serum-free medium, or FBS (10% (*v*/*v*)) for 24 h. Experiments with α-T-13′-COOH and CAY10566 were carried out in independent experiments. Fold changes (FCs) relative to the respective vehicle-treated control (w/o) are shown. Points represent data from six or seven independent biological replicates and their means ± standard error of the mean (SEM) are shown. *p*-Values were calculated using repeated measurement one-way ANOVA with Tukey’s post-hoc test. *, *p* < 0.05, **, *p* < 0.01.

To investigate the impact of α-T-13′-COOH on the proliferation of murine RAW264.7 macrophages, we determined the incorporation of 5-bromo-2-desoxyuridine (BrdU) into DNA over 24 h. α-T-13′-COOH at 5 µM significantly reduced the rate of BrdU incorporation, whereas lower concentrations (0.5 µM) were without effect (Figure 1).

**Figure 2:**
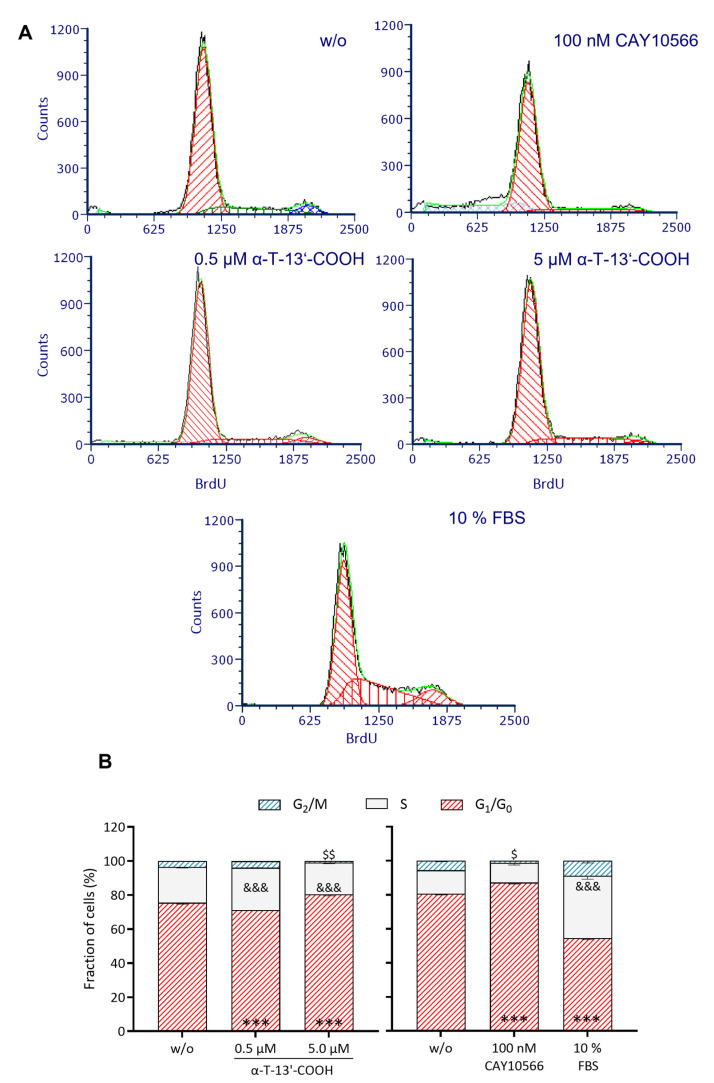
α-T-13′-COOH augments the proportion of cells in the G_1_/G_0_ phase. Cell cycle analysis (**A**) and quantification of each cell cycle phase (**B**) were performed by flow cytometry following propidium iodide staining. RAW264.7 cells were incubated with either vehicle (DMSO, ‘w/o’), α-T-13′-COOH (0.5 µM, 5.0 µM), the SCD1 inhibitor CAY10566 (100 nM), or FBS (10% (*v*/*v*)), which served as positive control. Studies with α-T-13′-COOH and CAY10566 were carried out in independent measurements. Data from five independent biological replicates are presented as means − SEM. Statistical significance was evaluated using repeated measurement two-way ANOVA with Tukey’s post-hoc test. ***, *p* < 0.001 (data of G_1_/G_0_ phase vs. w/o); &&&, *p* < 0.001 (data of S phase vs. w/o); $, *p* < 0.05; $$, *p* < 0.01 (data of G_2_/M phase vs. w/o).

The effects on cell cycle progression were investigated by propidium iodide staining and flow cytometry. α-T-13′-COOH at a low concentration (0.5 µM) increased the number of cells in the S phase (1.18-fold), indicating a slight stimulation of cell proliferation, and is also observed when stimulated with fetal bovine serum (FBS) (positive control) (Figure 2). In contrast, higher concentrations of α-T-13′-COOH (5 µM) resulted in the accumulation of cells in the G_0_/G_1_ phase (Figure 2). The exact mechanism by which α-T-13′-COOH induces G_0_/G_1_ cell cycle arrest requires further investigation.

### 2.2. α-T-13′-COOH Induces Apoptosis

**Figure 3:**
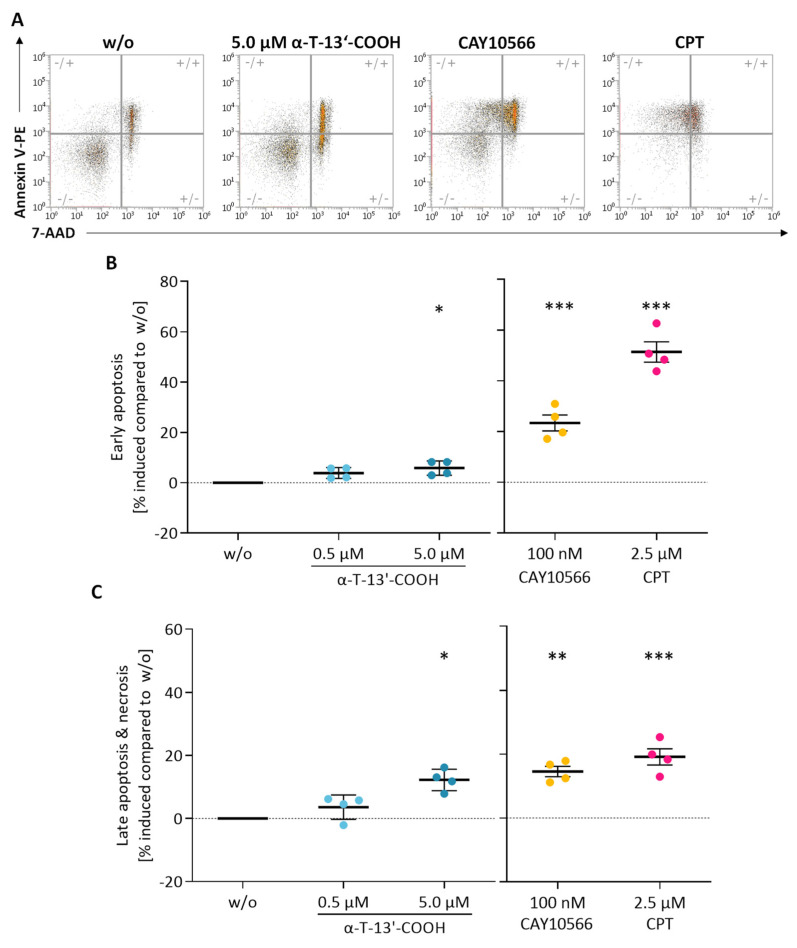
α-T-13′-COOH induces apoptosis in murine macrophages. Representative flow cytometry plots (**A**) and quantification of early apoptosis (**B**), late apoptosis, and necrosis (**C**) by staining with 7-AAD and PE-labelled Annexin V. RAW264.7 cells were cultivated with either vehicle (DMSO, ‘w/o’), α-T-13′-COOH (0.5 µM, 5 µM), the SCD1 inhibitor CAY10566 (100 nM), or the positive control camptothecin (CPT, 2.5 µM) for 24 h. Experiments with α-T-13′-COOH and CAY10566 were performed independently from each other. Relative changes in the number of apoptotic or necrotic cells compared to the vehicle control are shown. Means ± SEM of four or five independent experiments (dots) are shown. *p*-Values were calculated using repeated measurement one-way ANOVA with Tukey’s post hoc test. *, *p* < 0.05, **, *p* < 0.01; ***, *p* < 0.001 compared with w/o.

We then investigated the influence of α-T-13′-COOH on apoptosis and necrosis using 7-aminoactinomycin (7-AAD) and PE Annexin V staining in flow cytometric analyses (Figure 3A). α-T-13′-COOH induced both early (Figure 3B) and late apoptosis (Figure 3C) at a concentration of 5 µM. The topoisomerase I inhibitor camptothecin (CPT) was used as a positive control and efficiently induced apoptotic cell death as expected (Figure 3).

### 2.3. α-T-13′-COOH Modulates Expression of Stress-Related Genes

**Figure 4:**
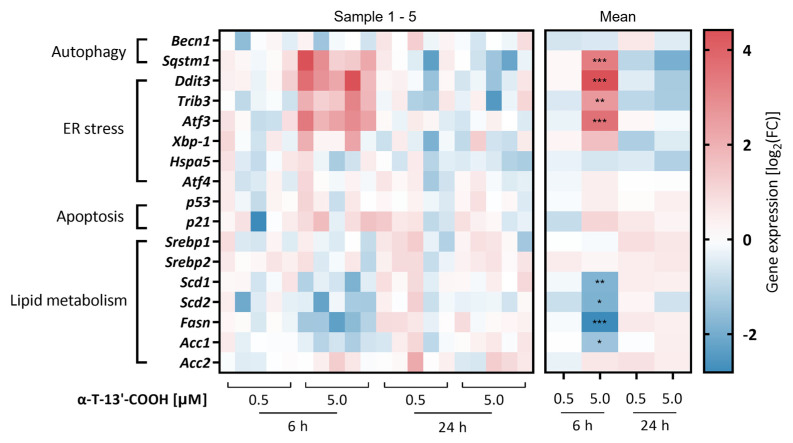
α-T-13′-COOH regulates the expression of genes involved in the modulation of lipid homeostasis, ER stress, autophagy, and apoptosis. Murine RAW264.7 macrophages were treated with vehicle (DMSO, ‘w/o’) or α-T-13′-COOH (0.5 µM, 5 µM) for the indicated periods (6 or 24 h). The level of the indicated mRNA was assessed by RT-qPCR and normalized to the expression of the reference gene peptidylprolyl isomerase B (Ppib), which is constantly expressed under all conditions. Fold changes (FC) were calculated vs. the respective vehicle controls. The log_2_ fold change (log_2_(FC)) is shown in the heatmap. Data from five independent biological replicates are presented as single data. Red color indicates higher expression and blue color indicates lower expression compared to vehicle control. Statistical comparisons were made by repeated measurement two-way ANOVA with Bonferroni’s post-hoc test between control and α-T-13′-COOH-treated samples. *, *p* < 0.05; **, *p* < 0.01; ***, *p* < 0.001. Abbreviations: Acc, acetyl-CoA carboxylase; Atf, cyclic AMP-dependent transcription factor; Becn, beclin; Ddit, DNA damage-inducible transcript; Fasn, fatty acid synthase; Hspa, heat shock 70 kDa protein; p21, cyclin-dependent kinase inhibitor 1; p53, tumor protein p53-inducible protein 3; Scd, stearoyl-CoA desaturase; Sqstm, sequestosome; Srebp, sterol regulatory element binding transcription factor; Trib, tribbles pseudokinase; Xbp-1, X-box binding protein 1.

To explore the anti-proliferative and pro-apoptotic mechanisms of α-T-13′-COOH, we first analyzed the mRNA expression of ten stress-related genes, with key roles in apoptosis, autophagy, and the unfolded protein response (UPR) (Figure 4). Most of the genes were either up-regulated or hardly affected by α-T-13′-COOH (5 µM) at 6 h, with the strongest effects on (i) sequestosome (Sqstm) 1, which is located at the crossroad of autophagy, the UPR, and stress-activated kinases [37,38], (ii) the C/EBP family member, DNA damage-inducible transcript (Ddit) 3, which is activated by ER stress and stimulates apoptosis [39], (iii) tribbles pseudokinase (Trib) 3, a putative protein kinase that participates in ER stress-dependent cell death induction via Ddit3 and regulates mitogenic and stress-activated kinases (MSKs) [40], and (iv) the stress-regulated transcription factor, cyclic AMP-dependent transcription factor (Atf) 3, which controls cell metabolism and immunity [41] (Figure 4). Together, the stress-regulatory genes involved in autophagy, UPR, and apoptosis are induced by α-T-13′-COOH before cell cycle arrest and apoptosis become apparent.

### 2.4. α-T-13′-COOH Decreases the MUFA/SFA Ratio in Triglycerides and Phospholipids

**Figure 5:**
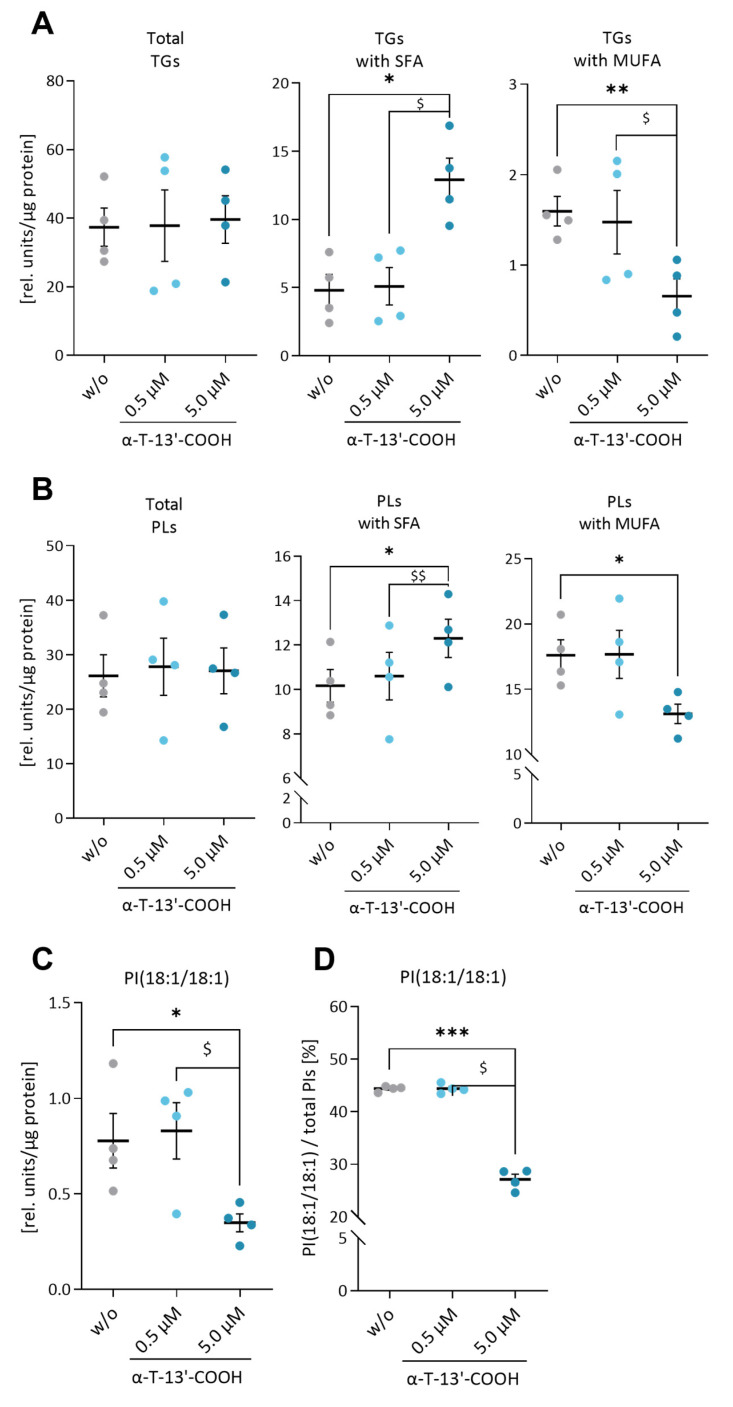
α-T-13′-COOH alters the lipid composition of triglycerides and phospholipids by increasing the amount of SFA-containing species and decreasing that of MUFA-containing species in murine macrophages. RAW264.7 cells were incubated with vehicle (DMSO, ‘w/o’) or 0.5 or 5.0 µM α-T-13′-COOH for 24 h. The content of different species of TGs and phospholipids was then analyzed by UPLC-MS/MS. (**A**–**C**) show the absolute content of lipid species in murine macrophages RAW264.7 ((**A**), triglycerides; (**B**), phospholipids; (**C**), PI(18:1/18:1)). The relative proportions of PI(18:1/18:1) in total PI are shown in (**D**). Experiments were performed in four independent biological replicates (dots). Data are presented as means ± SEM. *p*-Values were calculated using repeated measurement one-way ANOVA with Tukey’s post-hoc test. *, *p* < 0.05; **, *p* < 0.01; ***, *p* < 0.001 (vehicle control (w/o) vs. α-T-13′-COOH) or $, *p* < 0.05; $$, *p* < 0.01 (0.5 µM α-T-13′-COOH vs. 5.0 µM α-T-13′-COOH).

**Figure 6:**
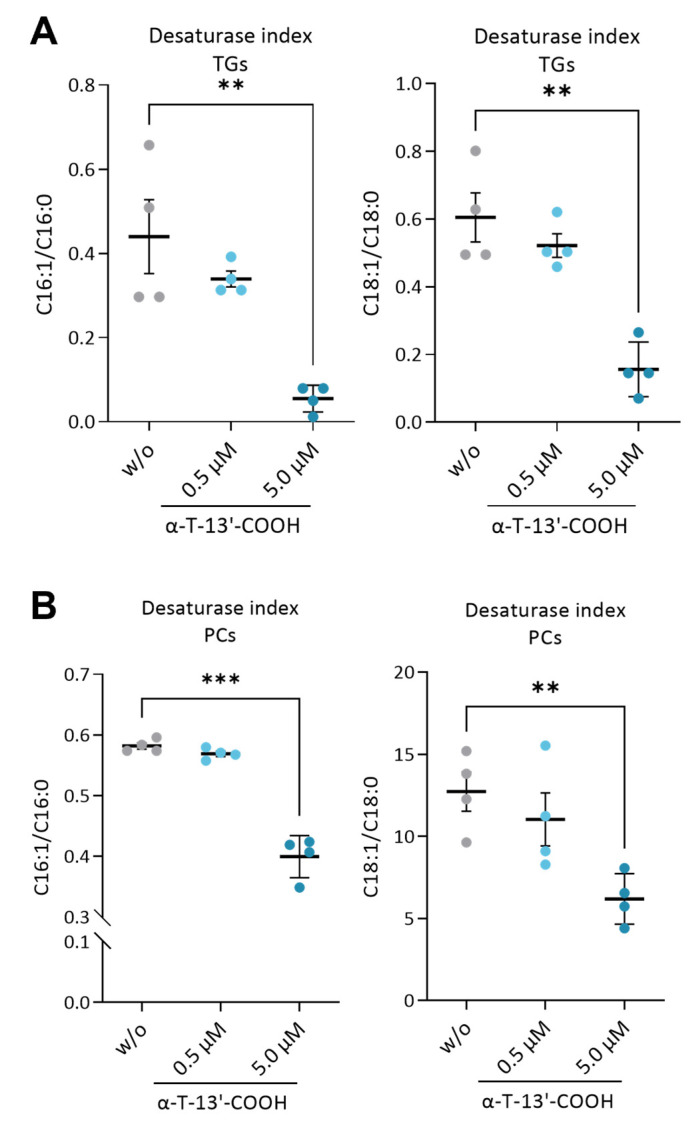
α-T-13′-COOH reduces the estimated enzyme activity of SCD1. The desaturation activity of SCD1 is estimated as the product-to-substrate ratios (C16:1/C16:0 and C18:1/C18:0) of lipid species ((**A**), TGs; (**B**), PC). Experiments were performed in four independent biological replicates (dots). Data are presented as means ± SEM. *p*-Values were calculated using repeated measurement one-way ANOVA with Tukey’s post-hoc test. **, *p* < 0.01; ***, *p* < 0.001; (w/o vs. α-T-13′-COOH).

Given the important role of lipid metabolism in balancing stress and survival signaling, including autophagy and UPR [28,42,43], we investigated the effect of α-T-13′-COOH on the cellular lipidome, focusing on triglycerides (TGs) and major membrane phospholipids, i.e., phosphatidylcholines (PCs), phosphatidylethanolamines (PEs), phosphatidylserines (PSs), phosphatidylinositols (PIs), and phosphatidylglycerols (PGs). α-T-13′-COOH did not substantially affect total TGs (Figure 5A) or total phospholipids levels within 24 h (Figure 5B), but at 5 µM, caused a strong shift from MUFA (C16:1; C18:1)- to SFA (C16:0; C18:0)-containing species across the studied lipid classes (Figure 5A,B, Supplementary Figures S3 and S4), as indicated by the drop in the desaturate index for TGs (Figure 6A) and PCs (Figure 6B).

The cellular MUFA/SFA ratio is adjusted by SCDs, which introduce a Δ9-cis double bond into saturated acyl-CoA esters [44]. The isoenzyme SCD1 maintains ER homeostasis, suppresses cell death programs, supports proliferation, and regulates stress-adaptive responses, including the UPR and autophagy [44]. Mechanistically, SCD1 inhibition lowers the cellular availability of the lipokine PI(18:1/18:1), which represses protein phosphatase 2A and triggers stress signaling and cell death initiation [37]. In fact, we found that both the absolute amount and the cellular proportion of PI(18:1/18:1) are strongly reduced in α-T-13′-COOH-treated RAW264.7 cells (Figure 5C,D), which might explain the cytotoxic and anti-proliferative activity of the vitamin E metabolite at micromolar concentrations.

### 2.5. α-T-13′-COOH Modulates Expression of Scd1 and Other Genes Encoding for Enzymes in De Novo Fatty Acid Biosynthesis

**Figure 7:**
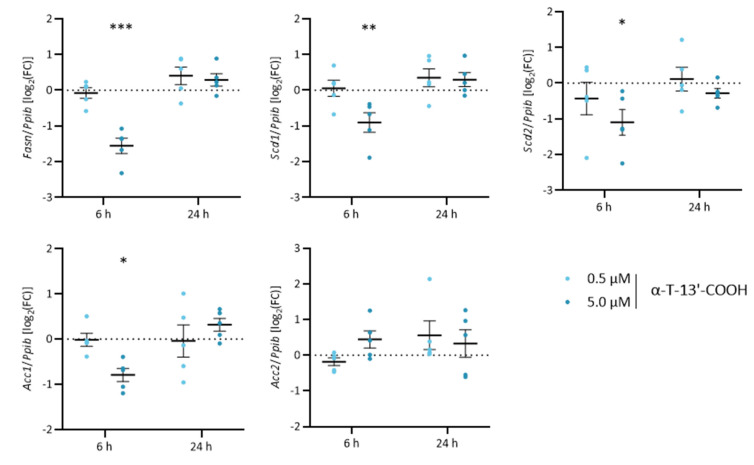
mRNA expression of genes involved in de novo lipogenesis of MUFAs is reduced by α-T-13′-COOH in murine RAW264.7 macrophages. The impact of α-T-13′-COOH on mRNA expression of Fasn, Scd1, Scd2, Acc1, and Acc2 was determined by RT-qPCR under serum-free conditions. RAW264.7 cells were incubated with either vehicle (DMSO, ‘w/o’) or 0.5 or 5.0 µM α-T-13′-COOH for the indicated times (6 or 24 h). Expression levels of target genes were normalized to the expression of the reference gene Ppib which was not affected. FCs were calculated vs. the respective vehicle-treated control and the log_2_(FC) is shown in the figure. Experiments were performed in five independent biological replicates (dots). The data are also shown as part of the heatmap in Figure 4. Data are presented as means ± SEM. Statistical comparisons were made by repeated measurement two-way ANOVA with Bonferroni’s post-hoc test between control and α-T-13′-COOH-treated samples. *, *p* < 0.05; **, *p* < 0.01; ***, *p* < 0.001.

To confirm our hypothesis that α-T-13′-COOH attenuates the cellular capacity to produce MUFAs, we analyzed the expression of MUFA-biosynthetic isoenzymes, i.e., acetyl-CoA carboxylase (Acc)1, Acc2, fatty acid synthase (Fasn), Scd1, and Scd2. α-T-13′-COOH (5 µM) significantly decreased the mRNA levels of these enzymes at 6 h, except for Acc2, whose expression was slightly induced (Figure 7). While Acc2 is functionally coupled to Fasn, Acc2-derived malonyl-CoA allosterically inhibits carnitine palmitoyltransferase-1, which transfers acyl-CoAs into the mitochondrial matrix for β-oxidation [45,46]. The counter-regulation of Acc1 and Acc2 might therefore indicate a switch from fatty acid biosynthesis to degradation.

**Figure 8:**
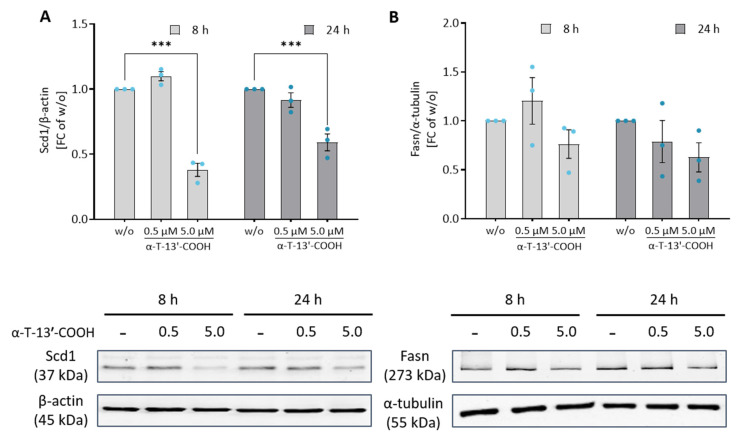
The amount of Scd1 protein is reduced by α-T-13′-COOH. The expression of Scd1 protein (≈37 kDa) was investigated by Western blotting of whole cell lysates. RAW264.7 macrophages were treated with either vehicle (DMSO, ‘w/o’) or 0.5 or 5.0 µM α-T-13′-COOH for 8 or 24 h. The signals of Scd1 and Fasn were normalized to the signal of the loading control β-actin (≈45 kDa) or α-tubulin (≈55 kDa), respectively. The graphs show the fold changes of Scd1 protein levels (**A**) or Fasn protein levels (**B**) in cells treated with α-T-13′-COOH relative to vehicle-treated control cells (w/o). Representative immunoblots are shown in the panels below each graph. Experiments were performed in three independent biological replicates (dots). Unprocessed Western blot images are shown in Supplementary Figures S5 and S6. Data are presented as means ± SEM. Statistical comparisons were made by repeated measurement two-way ANOVA with Tukey’s post-hoc test between control and α-T-13′-COOH-treated samples. ***, *p* < 0.001.

We next confirmed the effect of α-T-13′-COOH on Scd1 mRNA expression also at the protein level using an isoenzyme-specific antibody. α-T-13′-COOH at a concentration of 5 µM repressed Scd1 protein expression after 8 and 24 h (Figure 8A). In contrast, the protein levels of Fasn were only slightly reduced (Figure 8B), despite the more pronounced decrease in mRNA expression compared to Scd1 (Figure 4 and Figure 7), which might be explained by a slower turnover and longer half-life of the enzyme [47,48]. Together, α-T-13′-COOH reduces the mRNA expression of fatty acid biosynthetic enzymes, which results in the rapid and preferential decrease in Scd1 protein levels in macrophages.

### 2.6. SCD1 Repression Contributes to α-T-13′-COOH-Induced Cell Cycle Arrest and Apoptosis

**Figure 9:**
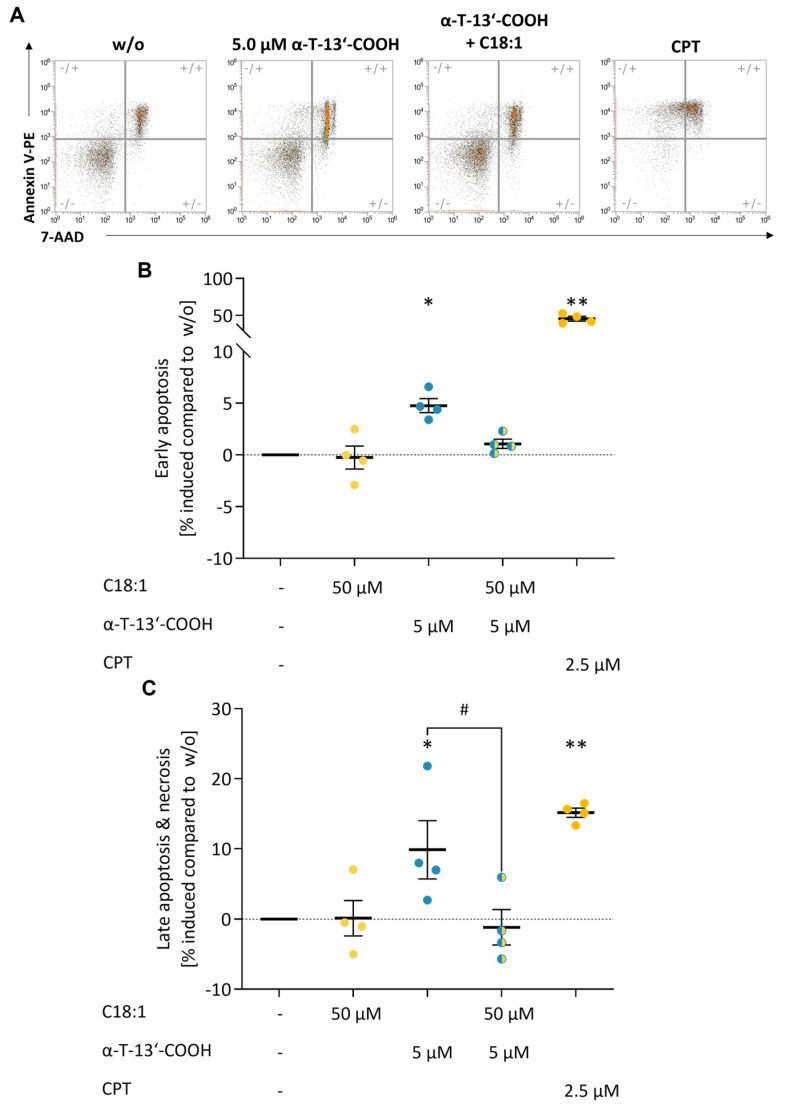
C18:1 prevents α-T-13′-COOH-induced apoptosis in murine macrophages. Representative flow cytometry plots (**A**) and quantification of early (**B**) and late (**C**) apoptosis using a dual staining with 7-AAD and PE Annexin V. RAW264.7 cells were treated with either α-T-13′-COOH (5.0 µM) or C18:1 (50 µM), the combination of 5.0 µM α-T-13′-COOH and 50 µM C18:1, or the positive control CPT (2.5 µM) for 24 h. Changes in the percentage of apoptotic or necrotic cells compared to the vehicle control are shown. Means ± SEM of four independent experiments (dots) are shown in (**B**,**C**). *p*-Values were calculated using repeated measurement one-way ANOVA with Tukey’s post-hoc test. Treated samples vs. vehicle control: *, *p* < 0.05; **, *p* < 0.01; α-T-13′-COOH vs. combined treatment: #, *p* < 0.05.

To explore the functional consequences of impaired Scd1 activity on cell cycle arrest and apoptosis, we incubated macrophages with the SCD1-specific inhibitor CAY10566. As observed for α-T-13′-COOH (Figure 1, Figure 2 and Figure 3), CAY10566 suppressed BrdU incorporation into DNA (Figure 1), elevated the fraction of cells in the G_0_/G_1_-phase (Figure 3), and increased the proportion of PE Annexin V and PE Annexin V/7-AAD positive cells (Figure 2). Compared to α-T-13′-COOH, CAY10566 led to a more pronounced apoptotic phenotype with a higher number of apoptotic cells, as would be expected from a direct inhibition of SCD1 (Figure 2). Next, we challenged macrophages with α-T-13′-COOH and supplemented the SCD1-derived C18:1, which is efficiently incorporated into PI(18:1/18:1) and other cellular lipids [28,37]. Consistent with our hypothesis, C18:1 partially prevented PS externalization (measured as the staining of PE annexin V) in the presence of α-T-13′-COOH (Figure 9). Thus, our data indicate that the decrease in Scd1 expression is vital for the induction of apoptosis by α-T-13′-COOH in macrophages and likely also mediates G_0_/G_1_ cell cycle arrest.

### 2.7. α-T-13′-COOH Inhibits the Proteolytic Activation of SREBP1

**Figure 10:**
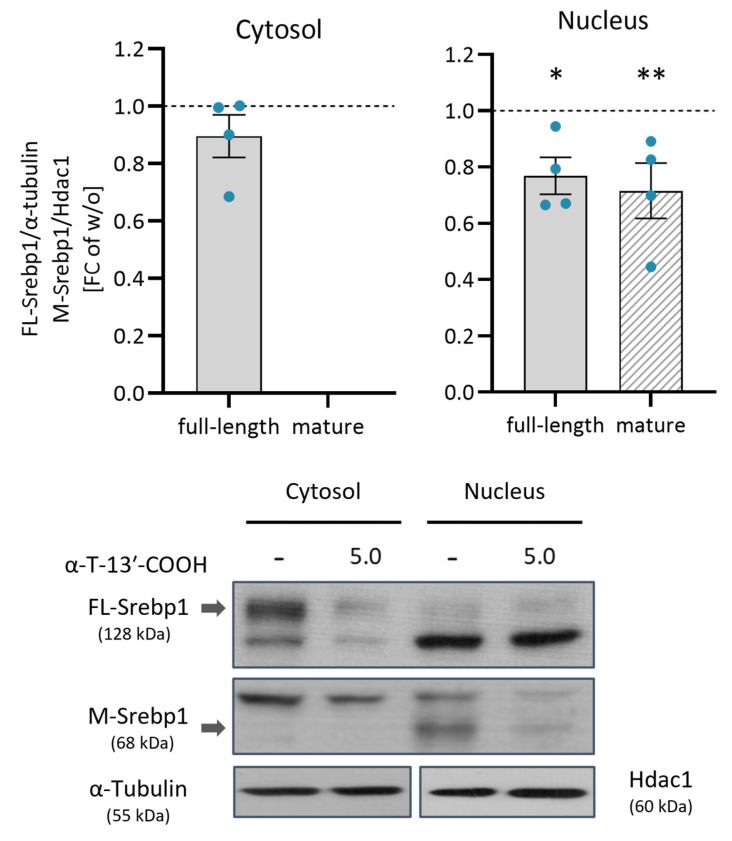
α-T-13′-COOH reduces the nuclear translocation of the transcription factor SREBP1. Murine RAW264.7 macrophages were treated with 5 µM α-T-13′-COOH for 12 h. The cytoplasm and nuclei of the cells were then isolated. The protein expression of Srebp1 (full-length approx. 128 kDa; mature (cleaved) approx. 68 kDa) was quantified by Western blotting of the separated cellular compartments. Srebp1 expression was normalized to the reference protein α-tubulin (cytosolic fraction) or Hdac1 (nuclear fraction). Graphs show fold changes of Srebp1 levels in cells treated with α-T-13′-COOH relative to vehicle-treated control cells (shown as dotted line). Representative immunoblots are shown in the panel below the graphs. An unprocessed Western blot image is shown in Supplementary Figure S7. Experiments were performed in four independent biological replicates (dots). Data are presented as means ± SEM. Statistical comparisons were made by paired two-tailed multiple *t*-tests between control and α-T-13′-COOH-treated samples. *, *p* < 0.05, **, *p* < 0.01.

Enzymes in fatty acid biosynthesis, including ACC, SCD1, and FAS, are under the control of the transcription factor SREBP1 [49,50,51], which enters the nucleus after being released by proteolytic cleavage from an inactive precursor located in the ER membrane [52]. α-T-13′-COOH (5 µM) substantially decreased the levels of mature Srebp1 (M-Srebp1), both in the cytoplasm and nucleus (Figure 10), whereas Srebp1 mRNA expression (Figure 4) and the cytoplasmic availability of the full-length protein (FL-Srebp1) were hardly affected (Figure 10). We conclude that the repression of Scd1 by α-T-13′-COOH depends, at least in part, on an impaired proteolytic conversion of FL-Srebp1 into the mature transcription factor (M-Srebp1).

## 3. Discussion

Vitamin E has been proposed to suppress cancer development [18,20,53], although the underlying molecular mechanisms are largely unknown. Here, we report that the endogenously formed α-TOH metabolite α-T-13′-COOH induces cell cycle arrest along with apoptosis by interfering with fatty acid desaturation in murine leukemic macrophages. The genes involved in (adaptive) stress responses, such as autophagy, UPR, and apoptosis, are induced in parallel, and cells are depleted of the stress-protective lipokine PI(18:1/18:1). Based on these results, we hypothesize that α-T-13′-COOH may contribute to the putative cancer-preventive properties of vitamin E.

Until the last decade, attention was paid to α-TOH and its potential anti-cancer properties based on the hypothesis that α-TOH could efficiently neutralize the oxidative stress implicated in the pathophysiology of many cancers [20]. Epidemiological and interventional studies conducted in humans with α-TOH failed to show the benefit of a supplementation of vitamin E [20,54]. Conversely, concerns have been raised about the safety of high-dose vitamin E supplementation (>400 IU/day) due to the potentially promoted cancer metastasis [55] and increased all-cause mortality observed in some vitamin E supplementation trials [56]. There is, so far, no reliable explanation for the inconsistent and conflicting results of the interventional studies on vitamin E and cancer risk. However, recent studies associated α-TOH with a protective role in the regulation of ferroptosis [57,58], which is an iron-dependent cell death program that relies on excessive membrane phospholipid peroxidation [32,59]. Of note, ferroptosis is considered a cancer-suppressive mechanism and a potential therapeutic strategy for treating multiple types of cancer, including therapy-resistant tumors [60], and has been linked to SREBP1 and SCD1 [30]. Thus, the regulation of ferroptosis by α-TOH may partially explain the inefficacy of vitamin E in controlling cancer progression.

Recent studies on vitamin E metabolism revealed a new perspective on the cancer-preventing potential of vitamin E. Galli et al. found that the terminal metabolite of the catabolism of γ-tocopherol, i.e., γ-carboxyethyl-hydroxychroman (γ-CEHC), strongly suppresses the proliferation of human prostate cells (83% ± 5% inhibition at the concentration of 50 µM), which might be attributed to an arrest of cell cycle progression [61]. Birringer et al. reported pro-apoptotic properties for the LCMs of α- as well as δ-TOH in HepG2 cells (EC_50_ = 6.5–13.5 µM). Mechanistically, α- and δ-13′-T-COOH (10 µM) cause mitochondrial dysfunction through oxidative damage and, consequently, cell death [15]. Distinct metabolites of tocotrienols (T3s) have a positive impact on the development of colon carcinoma: δ-T3-13′-COOH, a naturally occurring constituent of the seeds of *Garcinia kola*, limited colon tumor cancer growth in mice, which was correlated with the inhibition of the tumor-promoting enzymes cyclooxygenase-2 (COX-2) and 5-LO, and ascribed to the regulation of autophagy caused by aberrant (dihydro)ceramide and sphingomyelin metabolism in cancer cells [22].

SCD1 maintains cellular fatty acid and thus membrane homeostasis by converting SFA-CoA to MUFA-CoA. Accumulating evidence suggests a correlation between the abnormally enhanced proliferation of cancer cells and increased expression of SCD1 [34,44,62,63]. SCD1 is considered to fuel cancer cell proliferation, tumor growth, and metastasis [44]. We found that the cytotoxic effects of α-T-13′-COOH are essentially mediated by the suppression of SCD1-mediated MUFA biosynthesis. α-T-13′-COOH down-regulates the expression of SCD1 at the mRNA and protein levels, and lowers the proportion of MUFA while increasing that of SFA in cellular triglycerides and phospholipids. Major changes in lipid droplet size or number are not to be expected, although α-T-13′-COOH substantially changed the fatty acid composition of triglycerides, which together with cholesteryl esters, form the core of lipid droplets. On the one hand, we here show that 24 h treatment with α-T-13′-COOH does not alter the total triglyceride levels (Figure 5A). On the other hand, we have recently shown for THP-1 macrophages that lipid droplets are not altered by treatment with 5 µM α-T-13′-COOH for 24 h [6]. Notably, the supplementation of the SCD1 product C18:1 essentially abolished apoptosis induction by α-T-13′-COOH, which indicates that impaired fatty acid desaturation essentially contributes to the cytotoxic activity of the vitamin E metabolite.

Selective interference with SCD1 activity disturbs cellular lipid homeostasis and ER function, which in turn increases sensitivity to lipotoxicity and ferroptosis, induces adaptive stress signaling via p38 mitogen-activated protein kinases (MAPK), autophagy, and UPR, and ultimately triggers cell cycle arrest and initiates programmed cell death [64,65,66,67]. Interestingly, markers of autophagy (*Sqstm1*), ER stress (*Ddit3*, *Trib3*, *Atf3*, *Xbp-1*), and apoptosis (*p21*, *p53*) were also up-regulated by α-T-13′-COOH, prior to cytotoxic effects. To study the consequences of the altered lipid metabolism on cytotoxicity, we selectively inhibited SCD1 by CAY10566 and evaluated the effect on cell proliferation and programmed cell death. In support of our hypothesis, the direct inhibition of SCD1 mimicked the effects of α-T-13′-COOH caused by the down-regulation of Scd1 expression. Our findings are in line with several studies reporting the pro-apoptotic and anti-proliferative effects of CAY10566 in multiple cell lines in vitro and also in (tumor) tissues in vivo [37,67,68,69,70].

By converting SFA-CoA into MUFA-CoA (i.e., C16:1- and C18:1-CoA), SCD1 determines the saturation of membrane phospholipids and neutral lipids, such as triglycerides. Several mechanisms have been proposed to orchestrate the biological functions of SCD1 at the molecular level [28]. These speculations include the effects on biophysical membrane properties and microdomain structures, specific receptors, fatty acid membrane anchors, redox-protective functions, and MUFA-derived bioactive lipids [35,36,71,72]. Recent mechanistic insights suggest that the SCD1-derived lipokine PI(18:1/18:1) plays a central role in counteracting stress responses by inhibiting the activation of p38 MAPK, regulating autophagy, attenuating ER stress, the UPR, and apoptosis, while maintaining cell morphology and proliferation [37]. PI(18:1/18:1) is considered to be a stress sensor, which may enhance tolerance to (patho)physiological (metabolic) challenges that might otherwise culminate in programmed cell death. In fact, we here show that the cellular proportion of PI(18:1/18:1) is strongly decreased in leukemic macrophages in response to α-T-13′-COOH, and this drop in PI(18:1/18:1) is associated with the transcriptional regulation of adaptive stress pathways (including autophagy and the UPR) and enhanced apoptosis. Further supporting a functional link between SCD1 repression and cell death induction, C18:1 supplementation counteracts α-T-13′-COOH-induced cell death. Note that exogenous C18:1 is effectively incorporated into PI(18:1/18:1) and exerts stress protection through PI(18:1/18:1)-dependent and -independent mechanisms [37].

α-T-13′-COOH stimulates p38 MAPK activation in RAW264.7 cells at concentrations that induce cytotoxicity [11]. p38 MAPK is negatively regulated by SCD1/PI(18:1/18:1) [37,67] and in the control of pleiotropic stress responses, but also regulates homeostatic processes, including cell cycle progression, cell death, proliferation, and differentiation [73]. We therefore speculate that the SCD1-mediated depletion of MUFAs, together with enhanced p38 MAPK stress signaling, contributes to the cytotoxic activity of α-T-13′-COOH.

SREBP1 is a key regulator of de novo lipogenesis [26,28,74], and our study shows that α-T-13′-COOH decreases the proteolytic cleavage of SREBP1, thereby reducing the availability of the mature form that enters the nucleus and acts as a transcription factor [75]. In turn, the expression of lipogenic genes such as *Scd1*, *Fasn,* and *Acc1* is reduced. We therefore conclude that the attenuated proteolytic activation of SREBP1 and the associated repression of SCD1 is an important mechanism by which α-T-13′-COOH reduces MUFA biosynthesis. However, the existence of SREBP1-independent mechanisms cannot be excluded. Such mechanisms might involve transcription factors, such as liver X receptor (SREBP1-dependent and -independent), carbohydrate response element-binding protein, and peroxisome proliferator-activated receptors (PPARs), which tightly control fatty acid biosynthesis and degradation according to the cellular needs [28,74,76,77]. Still elusive is the mechanism by which α-T-13′-COOH suppresses the proteolytic activation of SREBP1. Among the major SREBP1-regulatory cascades to be addressed in future studies are PI3K/Akt/mTOR signaling toward SREBP1c expression, phosphorylation, and maturation [31,78,79], the cAMP/cAMP-dependent protein kinase A, which suppresses SREBP1 expression and maturation [80], and nuclear receptors/transcription factors that crosstalk with SREBP1, including liver X receptor, PPARs, peroxisome proliferator-activated receptor γ coactivator 1 (PGC-1), signal transducer and activator of transcription (STAT), and Myc [81,82,83,84]. Epigenetic modification by histone acetyltransferases and sirtuins [85], as well as sterols, oxysterols, and PUFAs [52,86], further control SREBP1 expression, maturation, and/or translocation. Overall, SREBP1 is a central hub in the coordination of lipid metabolism that is tightly regulated by a multitude of pathways, whose global deciphering demands for multiomics approaches that are in progress.

## 4. Materials and Methods

### 4.1. Materials

#### 4.1.1. Chemicals

All chemicals were obtained from either Carl Roth (Karlsruhe, Germany), Sigma-Aldrich (Darmstadt, Germany), Thermo Fisher Scientific (Darmstadt, Germany), Merck Millipore (Darmstadt, Germany), or Cayman Chemical (Ann Arbor, MI, USA), if not indicated otherwise.

#### 4.1.2. Semi-Synthesis of Vitamin E Metabolites and Concentration Determination

α-T-13′-COOH was obtained from the semi-synthesis of garcinoic acid (i.e., δ-T3-13′-COOH), which was isolated from the African bitternut *Garcinia kola*, as described [14,15]. The purity of α-T-13′-COOH of >95% was confirmed by ultrahigh-performance liquid chromatography (UHPLC) coupled with mass spectrometry (MS) [6]. Stock solutions of α-T-13′-COOH were prepared in dimethyl sulfoxide (DMSO), and the concentration of α-T-13′-COOH was determined prior to experiments by photometric absorption at 292 nm with a FLUOstar Omega microplate reader (BMG Labtech, Ortenberg, Germany). Depending on the concentration of the stock solution of α-T-13′-COOH, the same amount of DMSO that was in the α-T-13′-COOH-treated samples was added as a solvent control in all experiments.

### 4.2. Cell Culture

Murine RAW264.7 macrophages were obtained from the American Type Culture Collection (ATCC; Manassas, VI, USA). Cells were cultivated in 150 cm^2^ cell culture flasks (TPP Techno Plastic Products, Trasadingen, Switzerland) in high-glucose (4500 mg/L) Dulbecco’s Modified Eagle Medium (DMEM) containing 0.1 mg/mL penicillin/streptomycin/*L*-glutamine (PSG) and 10% (*v*/*v*) FBS. As recommended by the ATCC, cells were sub-cultured at about 80% confluence by detaching with a cell scraper and culturing with a mixture of 70% high-glucose DMEM (supplemented with PSG and FBS) and 30% culture medium, which was obtained from the previous passage [4]. Cells were kept at 37 °C in a humidified 5% CO_2_/95% (*v*/*v*) air atmosphere. For further experiments, harvested cells were seeded at different densities, cultured for another 24 h, and incubated with compounds for the times indicated.

### 4.3. Colorimetric Cell Proliferation Assay

The method of Hawker et al. [87] was adapted to measure cell proliferation. Briefly, RAW264.7 macrophages were seeded in a 48-well plate (TPP) at a density of 3 × 10^5^/well. Monolayers were serum-starved for another 24 h before treatment. High-glucose DMEM culture medium supplemented with 10% (*v*/*v*) FBS in DMEM was used as the control. BrdU was added to the incubation medium in a concentration of 10 µM. At the end of incubation, cells were washed with a non-supplemented medium and fixed with 70% (*v*/*v*) ethanol. Then, cells were disrupted and biomolecules denatured with 2 M HCl. After neutralization with borate buffer (0.1 M, pH 9), the cells were blocked with blocking buffer (2% (*v*/*v*) goat serum, 0.1% (*v*/*v*) Triton-X-100, 0.01% (*v*/*v*) thimerosal in phosphate-buffered saline pH 7.4, PBS). After 15 min at 37 °C, the monoclonal anti-BrdU antibody (1 µg/mL; Sigma-Aldrich) was added to each well, and incubation continued for 60 min. For the colorimetric detection, the wells were incubated with tetramethylbenzidine substrate (product-no. T0440, Sigma-Aldrich) until a blue color developed. The reaction was stopped after 30 min by adding 2 M H_2_SO_4_, and then the absorbance was measured from 450 nm to 570 nm with a FLUOstar Omega microplate reader (BMG Labtech).

### 4.4. Flow Cytometric Analysis of Cell Cycle

Cell cycle progression was determined through staining with propidium iodide solution and flow cytometric analysis, as described [88] Klicken oder tippen Sie hier, um Text einzugeben.. Cells were seeded at a density of 8 × 10^5^/well in a 12-well plate (TPP). High-glucose DMEM with 10% (*v*/*v*) FBS was used as the control. After the indicated treatment, cells were scraped in PBS (pH 7.4) and then fixed by 70% (*v*/*v*) ethanol for at least four hours. After fixation, cells were washed twice with ice-cold PBS, centrifuged at 400× *g* for 5 min, and the pelleted cells were stained in PI staining solution (0.1% (*v*/*v*) Triton X-100, 10 μg/mL PI, and 100 μg/mL deoxyribonuclease-free ribonuclease A in PBS) for 10 min at 37 °C. Flow cytometric analysis (excitation/emission of propidium iodide bound to DNA: ~535 nm/~615 nm) was performed with an Attune NxT flow cytometer (Thermo Fisher Scientific). At least 30,000 events were collected for a single measurement and residual debris were excluded by our gating strategy (Supplementary Figure S1). Data on cell cycle distribution were analyzed by using FCS Express version 7.08.0018 (De novo Software, Pasadena, CA, USA).

### 4.5. Flow Cytometric Analysis of Apoptosis and Necrosis

Apoptosis and necrosis were analyzed with the PE Annexin V Apoptosis Detection Kit I (BD Pharmingen, Heidelberg, Germany). First, 8 × 10^5^ cells per well of a 12-well plate were seeded in 3 mL DMEM, and 24 h after seeding, cells were treated with vehicle (DMSO), α-13′-COOH, or 2.5 µM CPT as the positive control. After another 24 h, culture supernatants were removed, and cells were washed with warm PBS and detached by scraping. CPT-treated samples were heated further at 55 °C for 30 min to induce necrosis. Other samples were washed twice with PBS and centrifuged at 400× *g* for 5 min. Cell pellets were resuspended in binding buffer at a concentration of 1 × 10^6^ cells/mL. For labeling, Annexin V and 7-AAD were added to the cell suspension, and samples were incubated at room temperature. After 15 min, samples were analyzed using an Attune NxT flow cytometer (Thermo Fisher Scientific). At least 20,000 events were collected for a single measurement and residual debris were excluded by our gating strategy (Supplementary Figure S2). The results were analyzed using Attune NxT Software Version 3.1 (Thermo Fisher Scientific). Annexin V-PE positive and 7-AAD negative cells represent early-stage apoptotic cells, whereas annexin V-PE and 7-AAD positive cells are found in late apoptosis with apoptotic and necrotic characteristics.

### 4.6. Analysis of mRNA Expression

#### 4.6.1. RNA Isolation and cDNA Synthesis

Total RNA was extracted using the RNeasy Mini kit (Qiagen, Hilden, Germany), as described [89]. Genomic DNA was removed by on-column DNase I digestion (Qiagen). cDNA synthesis was performed using a Revert Aid First Strand cDNA Synthesis kit (Fermentas, St. Leon-Rot, Germany) with 5 µg total RNA, 500 ng/µL oligo(dT) primer (Invitrogen, Carlsbad, CA, USA), 20 units ribonuclease inhibitor (Promega, Mannheim, Germany), 1 mM deoxyribonucleoside triphosphates (dNTPs, Fermentas), and 200 units M-MuLV reverse transcriptase RevertAid (Fermentas), as described in [90].

#### 4.6.2. Quantitative Real-Time Reverse Transcription PCR (RT-qPCR)

RT-qPCR was performed using Maxima SYBR Green qPCR Master Mix (Thermo Fisher Scientific), as described [4]. Each sample was measured in duplicates on a LightCycler 480 instrument (Roche Diagnostics, Mannheim, Germany). The cycling parameters included an initial incubation for 15 min at 95 °C, a 40-cycle PCR that consisted of denaturation at 94 °C for 15 s, and a combined annealing and extension phase at 60 °C for 30 s. Using the Light Cycler software version 1.5.0.39, the results were analyzed with the ΔΔCt method. PCR efficiency was tested using cDNA dilutions. All primers were purchased from Invitrogen (Thermo Fisher Scientific) and the sequences are given in Supplementary Table S1.

### 4.7. Lipidomics Analysis

#### 4.7.1. Lipid Extraction

Lipids were extracted from RAW264.7 cell pellets by the successive addition of methanol, PBS (pH 7.4), chloroform, and saline (final ratio: 34:14:35:17) [91,92]. After the evaporation of the organic solvent, the remaining lipid fraction was dissolved in methanol, stored at −20 °C, and analyzed by UPLC-MS/MS. Internal standards: 1,2-dimyristoyl-*sn*-glycero-3-phosphatidylcholine (DMPC), 1,2-dimyristoyl-*sn*-glycero-3-phosphatidyl-ethanolamine (DMPE) (Avanti Polar Lipids, Alabaster, AL, USA).

#### 4.7.2. Analysis of Phospholipid and TG Profiles by UPLC-MS/MS

Phospholipids (PC, PE, PI, PS, PG) were separated on an Acquity UPLC BEH C8 column (130 Å, 1.7 μm, 2.1 × 100 mm; Waters, Milford, MA, USA) using an Acquity UPLC system (Waters), which was coupled to a QTRAP 5500 mass spectrometer (Sciex, Framingham, MA, USA) equipped with a Turbo V Ion Source and an electrospray ionization probe [67,93,94]. Chromatographic separation was performed at a flow rate of 0.75 mL/min and at a column temperature of 45 °C. The mobile phase was composed of eluent A (acetonitrile/water, 95/5, with 2 mM ammonium acetate) and eluent B (water/acetonitrile, 90/10, with 2 mM ammonium acetate). The gradient was ramped from 70% to 80% A within 5 min and to 100% A within 2 min, followed by isocratic elution for another 2 min. Eluted phospholipids were detected upon the fragmentation of parental ions (PC: [M+OAc]^−^, all other phospholipids: [M-H]^−^) to fatty acid anions derived from *sn*-1 and *sn*-2 positions by multiple reaction monitoring using a QTRAP 5500 mass spectrometer. The ion spray voltage was set to −4500 V, the curtain gas to 30 psi, the collision gas to medium, and the heated capillary temperature to either 350 °C (PC), 500 °C (PI), 550 °C (PS, PG), or 650 °C (PE). The sheath gas pressure was set to 45 (PS) or 55 psi (PC, PE, PI, PG) and the auxiliary gas pressure was set to either to 75 psi (PC, PE, PI, PG) or 80 psi (PS). The declustering potential was set to −40 V (PS), −44 V (PC), −45 V (PG), or −50 V (PE, PI), the entrance potential to −10 eV (PC; PE, PI, PS, PG), the collision energy to −38 eV (PE), −46 eV (PC), −52 eV (PG), −56 eV (PS), or −62 eV (PI), and the collision cell exit potential to −11 V (PC, PI), −12 V (PE), −18 V (PG), or −20 V (PS).

TGs were separated on an Acquity UPLC BEH C8 column (130 Å, 1.7 μm, 2.1 × 100 mm; Waters) using either an Acquity UPLC system (Waters), which was coupled to a QTRAP 5500 mass spectrometer (Sciex) equipped with a Turbo V Ion Source and an electrospray ionization probe [92], or an ExionLC AD UHPLC system (Sciex), which was coupled to a QTRAP 6500^+^ mass spectrometer (Sciex) equipped with an IonDrive Turbo V Ion Source and an electrospray ionization probe [95]. In brief, both LC systems were operated at a flow rate of 0.75 mL/min and a column temperature of 45 °C. The mobile phase consisted of eluent A (acetonitrile/water, 95/5, with 2 mM ammonium acetate) and eluent B (isopropanol). For the separation of TGs, eluent A was reduced from 90% to 70% within 6 min followed by isocratic elution for 4 min. The fragmentation of [M + NH_4_]^+^ adduct ions to [M-fatty acid anion]^+^ ions was measured by multiple reaction monitoring. The ion spray voltage was set to 5500 V, the curtain gas to 30 psi (QTRAP 5500) or 40 psi (QTRAP 6500^+^), the collision gas to low, and the heated capillary temperature to 400 °C. The sheath gas pressure was set to 60 psi and the auxiliary gas pressure was set to 70 psi. The declustering potential was set to 120 V, the entrance potential to 10 V, the collision energy to 35 eV, and the collision cell exit potential to 26 V.

Absolute lipid intensities were normalized to an internal standard (DMPC for PC, PI, PS, PG, and TG or DMPE for PE) and the protein amount. Relative lipid intensities represent the signals of individual lipid species given as a percentage of all the analyzed lipid signals within the subgroup (=100%). The absolute lipid intensities with SFA or MUFA in respective lipid subclasses were carried out by summary of the SFA or MUFA-containing lipid species, which are listed in Supplementary Table S2.

The instruments were either operated with Analyst 1.6.2 (QTRAP 5500, Sciex) or Analyst 1.7.1 (QTRAP 6500^+^, Sciex). Acquired extracted chromatograms were either processed with Analyst 1.6.2 or Analyst 1.6.3 (both Sciex).

#### 4.7.3. Determination of the TG and PC Desaturase Index

To estimate the activity of SCD1, we calculated desaturase indices separately for TG and PC based on the absolute data of the species that were analyzed by UPLC-MS/MS. The indices were defined as the product-to-substrate ratios C16:1 to C16:0 and C18:1 to C18:0 in the respective lipid subclass (Supplementary Table S2).

### 4.8. Immunoblotting

#### 4.8.1. Sample Preparation

Cell lysates: Cells were harvested using a non-denaturing buffer (50 mM Tris-HCl, 0.5% (*v*/*v*) Nonidet P40, 250 mM NaCl, 15 mM EDTA, 50 mM NaF, 0.5 mM Na_3_VO_4_) containing 1% (*v*/*v*) protease inhibitor and mixed 3:1 with SDS sample buffer (6.26% 1 M Tris-HCl, 2.3% (*w*/*v*) SDS, 10% glycerol (*v*/*v*), 5% (*v*/*v*) 2-mercaptoethanol, 0.1% (*w*/*v*) bromophenol blue).

Nuclear fractions: The NE-PER Nuclear and Cytoplasmic Extraction reagents (Thermo Fisher Scientific) were used to separate nuclear and cytoplasmic fractions for the study of SREBP1 translocation. Briefly, 2 × 10^6^ cells were harvested in 500 µL PBS and collected by centrifugation (400× *g*, 5 min). The supernatant was removed, 200 µL ice-cold CER I buffer was added to each pellet, and the sample was vigorously mixed for 15 s. After incubation on ice for 10 min, 11 µL ice-cold CER II buffer was added to the lysate. Samples were incubated for 1 min at 4 °C and mixed again for 15 s. To isolate the cytoplasmatic fraction, the lysate was centrifuged for 5 min at 16,000× *g* and the supernatant containing the cytoplasmic extract was transferred immediately to another tube. The insoluble fraction, which contains nuclei, was resuspended in 100 µL NER ice-cold buffer and incubated for 40 min on ice, interrupted by vigorous mixing for 15 s every 10 min. Finally, samples were centrifuged for 10 min at 16,000× *g* and the supernatant containing the nuclear fraction was collected. All samples were stored at −80 °C until Western blot analysis.

#### 4.8.2. SDS-PAGE and Western Blot Analysis

Proteins (10–20 µg) were separated by SDS-PAGE using a 10% (*v*/*v*) acrylamide gel and transferred to a PVDF membrane (VWR, Darmstadt, Germany) or a Hybond ECL nitrocellulose membrane (GE Healthcare, Munich, Germany) with 0.25 M Tris, 1.92 M glycine, 0.1% (*w*/*v*) SDS, and 20% (*v*/*v*) methanol (pH 8.3) or 0.04 M Tris-HCl, 0.04 M glycine, and 20% (*v*/*v*) methanol as the transfer buffer. The membranes were blocked with 5% BSA solution for 1 h at room temperature and then incubated with primary antibodies for 1 h at room temperature or overnight at 4 °C. Primary antibodies were directed against murine Scd1 (1:1000; rabbit anti-Scd1; #2438, Cell Signaling, Frankfurt am Main, Germany), Fasn (1:1000, rabbit anti-Fasn; #3189, Cell Signaling), Srebp1 (1:200 to 1:400, mouse anti-Srebp1; sc-13551, Santa Cruz Biotechnology, Heidelberg, Germany), Hdac1 (1:1000, mouse anti-Hdac1; sc-81598, Santa Cruz Biotechnology), β-actin (1:1000, mouse anti-β-actin; #3700, Cell Signaling), and α-tubulin (1:5000, mouse anti-α-tubulin; clone B-5-1-2, BD Biosciences, Heidelberg, Germany). The membranes were washed and incubated for 1 h with IRDye 800CW-labeled, 680LT-labeled, or HRP-coupled secondary antibodies, which were purchased from LI-COR Biosciences (Lincoln, NE) or DAKO (Hamburg, Germany). The SignalBoost Immunoreaction Enhancer kit (Calbiochem, Darmstadt, Germany) was used to enhance the chemiluminescence signals of Scd1 and Srebp1, while the antibodies against α-tubulin and Hdac1 were incubated in a hybridization buffer containing 0.5% (*w*/*v*) milk powder in PBS. The Pierce ECL Western Blotting Substrate (Thermo Fisher Scientific) was used for the detection of protein Srebp1, Hdac1, and α-tubulin. The fluorescence signals of the proteins Fasn, Scd1, and β-actin were recorded using an Odyssey infrared imager (LI-COR Biosciences, Lincoln, NE, USA).

To further validate the specificity of the mouse anti-Srebp1 antibody in RAW 264.7 cells, we induced Srebp1 expression using the LXR agonist T0901317 (product no. T2320, Sigma-Aldrich) for either 24 or 48 h, and then analyzed Srebp1 expression by Western blot (primary antibody dilution of 1:200) (Supplementary Figure S8).

### 4.9. Data Presentation and Statistical Analysis

Data are presented as either single data (Figure 4) or means ± SEM of independent experiments, as indicated in the figure legend. To test for the statistical significance of the differences of the data shown in Figure 1, Figure 3, Figure 5, Figure 6 and Figure 9, repeated measurement one-way ANOVA with Tukey’s post-hoc test was used. To test the statistical significance of the differences in the data shown in Figure 2, Figure 7 and Figure 8, repeated measurement two-way ANOVA with Tukey’s post-hoc test was used. To test the statistical significance of the differences in the data shown in Figure 10, a paired two-tailed multiple t-test was used. The calculations and presentation of data were performed using Prism version 9.0.0 (Graphpad, San Diego, CA, USA).

## 5. Conclusions

Here, we show that α-T-13′-COOH, an endogenous metabolite of α-TOH, induces cell cycle arrest and apoptosis in leukemic macrophages. Mechanistically, α-T-13′-COOH interferes with the proteolytic activation of the transcription factor SREBP1 and suppresses SCD1 expression. As a consequence, α-T-13′-COOH causes a shift from MUFA to SFA in major phospholipid classes and TGs, and decreases the cellular amount of the stress-protective signaling lipid PI(18:1/18:1). Our study provides insights into the tumor-suppressive mechanism of α-T-13′-COOH, thereby adding to the complexity that may underlie the mixed outcomes of α-TOH supplementation on cancer risk. Whether α-T-13′-COOH might also serve as a starting point for the development of multi-target anti-tumoral agents requires further investigations, including studies on patient-derived cancer cells and (leukemic) cancer models.

## Data Availability

All data are available from the corresponding authors on reasonable request.

## Supplementary Materials

The supporting information can be downloaded at: https://www.mdpi.com/article/10.3390/ijms24119229/s1.
