# Supplementary material for: α-Tocopherol-13′-Carboxychromanol Induces Cell Cycle Arrest and Cell Death by Inhibiting the SREBP1-SCD1 Axis and Causing Imbalance in Lipid Desaturation
Source: Int J Mol Sci. 2023 May 25;24(11):9229. doi: 10.3390/ijms24119229 (PMC10253185; doi:10.3390/ijms24119229)
Supplement: Supplementary file 1 [file ijms-24-09229-s001.zip › ijms-2379654-supplementary.pdf]

Supplementary Material

α-Tocopherol-13'-carboxychromanol induces cell cycle arrest and cell death by inhibiting the SREBP1-SCD1 axis and causing imbalance in lipid desaturation

Supplementary Table S1: Sequences of PCR primers.

Supplementary Table S2: SFA- or MUFA-containing lipid species in detected lipid subclasses.

Supplementary Figure S1: Additional measurement information of apoptosis and necrosis analysis.

Supplementary Figure S2: Additional measurement information of cell cycle analysis.

Supplementary Figure S3: Absolute amounts of SFA- or MUFA-containing species of phospholipids.

Supplementary Figure S4: Detected species of triglycerides and phospholipids.

Supplementary Figure S5: Unprocessed Western blot images of Scd1 protein.

Supplementary Figure S6: Unprocessed Western blot images of Fasn protein.

Supplementary Figure S7: Unprocessed Western blot images of Srebp1 protein.

Supplementary Figure S8: Validation of the specificity of the mouse anti-Srebp1 antibody in RAW264.7 macrophages.

Supplementary Table S1: Sequences of PCR primers used in the study. In each case, forward and reverse primers are located in different exons.

mRNA	mRNA name	Origin	GenBank accession No.	Forward primer	Reverse primer	Application size (bp)
Scd1	Stearoyl-CoA desaturase 1	Mus Musculus	NM_009127.4	CAGGTTTCCAAGCGCAGTTC	GAACTGGAGATCTCTTGGAGCA	144
Scd2	Stearoyl-CoA desaturase 2	Mus Musculus	NM_009128.2	TTAGCTCTCGGGAGAACATCTTG	GTTGATGTGCCAGCGGTACT	116
Fasn	Fatty acid synthase	Mus Musculus	NM_007988.3	GACTCGGCTACTGACACGAC	CGAGTTGAGCTGGGTTAGGG	123
Acc1	acetyl-Coenzyme A carboxylase alpha	Mus Musculus	NM_133360.3	CCGAGAAAGCAGGGGATCTG	TACCCGACGCATGGTTTTCA	91
Acc2	acetyl-Coenzyme A carboxylase beta	Mus Musculus	NM_001403527.1 NM_133904.3 NM_001403528.1 NM_001403529.1	CCGCTCAAGATCGAGGAGTC	TGATCTTAACCTCGGCCTGC	94
Becn1	Beclin 1	Mus Musculus	NM_019584.4 NM_001359819.1 NM_001359820.1 NM_001359821.1	GAACTCTGGAGGTCTCGCTC	AGCTCGTGTCCAGTTTCAGAG	180
Sqstm1	Sequestosome 1	Mus Musculus	NM_011018.3 NM_001290769.1	TTCGGAAGTCAGCAAACCTGA	CCGACTCCATCTGTTCCTCTG	116
Ddit3	DNA damage inducible transcript 3	Mus Musculus	NM_007837.4 NM_001290183.1	CCCAGGAAACGAAGAGGAAGA A	CGATGGTGCTGGGTACACTT	438
Ddit4	DNA damage inducible transcript 4	Mus Musculus	NM_029083.2	TCTTGTCCGCAATCTTCGCT	GGAGGACGAGAAACGATCCC	127
Trib3	Tribbles homolog 3	Mus Musculus	NM_175093.2	TTGTCTTGCGCGACCTCAA	AGTCATCACGCAGGCATCTT	98
Xbp-1	Spliced X-box binding protein 1	Mus Musculus	NM_001271730.1 NM_013842.3	AACAGAGTAGCAGCGCAGAC	GCCGTGAGTTTTCTCCCGTA	138
Hspa5	Heat shock 70kDa protein 5	Mus Musculus	NM_001163434.1 NM_022310.3	CACCTATTCCTGCGTCGGT	ATTCCAAGTGCGTCCGATGA	202
Atf4	Activating transcription factor 4	Mus Musculus	NM_001287180.1 NM_009716.3	AGTGGCATCTGTATGAGCCC	TCATAAGGTTTGGGCCGAGG	137
p53	Tumor protein p53-inducible protein 3	Mus Musculus	NM_011640.3	ACCTGCACAAGCGCCTCTC	CAGCTCCCGGAACATCTCG	105
p21	Cyclin-dependent kinase inhibitor 1	Mus Musculus	NM_001111099.2	TCTGGTGTCTGAGCGGCC	TCTGCGCTTGGAGTGATAGAAAT	106
Srebp1	Sterol regulatory element binding transcription factor 1	Mus Musculus	NM_011480.4 NM_001313979.1 NM_001358314.1 NM_001358315.1	GCGTGGTTTCCAACATGACC	TGTAGTGCCTCCTTTGCCAC	190
Srebp2	Sterol regulatory element binding transcription factor 2	Mus Musculus	NM_033218.1	GGCTGTCGGGTGTCATGG	TGACAAACTGTAGCATCTCGTCG	196

Supplementary Table S2: SFA- or MUFA-containing lipid species detected in the lipid subclasses PC, PI, PE, PS, PG and TG. The absolute intensities of PC, PI, PE, PS, PG and TG with SFA or MUFA species shown in **Fig. 3** and **Supplementary Fig. S3** were calculated by summarizing the absolute intensities of individual lipid species.

PCs		PIs		PEs	
SFA	MUFA	SFA	MUFA	SFA	MUFA
14:0_16:0	16:1_16:1	16:0_16:0	16:1_18:1	16:0_16:0	16:1_18:1
16:0_16:0	16:1_18:1	16:0_18:2	18:1_18:1	16:0_18:2	18:1_18:1
16:0_18:2	18:1_18:1	18:0_18:2	18:1_18:2	18:0_18:2	18:1_18:2
18:0_18:2	18:1_20:1	16:0_20:3	18:1_20:1	16:0_20:4	18:1_20:4
16:0_20:3	16:1_22:1	16:0_20:4	18:1_20:3	18:0_20:4	18:1_22:4
16:0_20:4	18:1_20:3	18:0_20:2	18:1_20:4	16:0_22:5	18:1_22:5
18:0_20:4	18:1_20:4	18:0_20:3	18:1_20:5	16:0_22:6	18:1_22:6
16:0_22:5	18:1_20:5	18:0_20:4		18:0_22:5	
16:0_22:6	18:1_22:4	16:0_22:5		18:0_22:6	
18:0_22:5	18:1_22:5	16:0_22:6			
18:0_22:6	18:1_22:6	18:0_22:5			
		18:0_22:6			

PSs		PGs		TGs	
SFA	MUFA	SFA	MUFA	SFA	MUFA
16:0_16:0	16:1_18:1	16:0_18:2	16:1_18:1	16:0_16:0_16:0	18:1_16:1_16:1
18:0_18:2	18:1_18:1		18:1_18:1	16:0_16:0_18:2	18:1_16:1_18:2
16:0_20:4	18:1_20:1		18:1_20:4	16:0_16:0_18:0	18:1_16:1_22:6
18:0_20:3	16:1_22:1		18:1_22:5	16:0_16:0_20:4	
18:0_20:4			18:1_22:6	16:0_18:0_18:2	
16:0_22:5				16:0_18:0_18:0	
16:0_22:6				16:0_16:0_22:6	
18:0_22:5				16:0_16:0_22:5	
18:0_22:6				18:0_18:2_18:2	

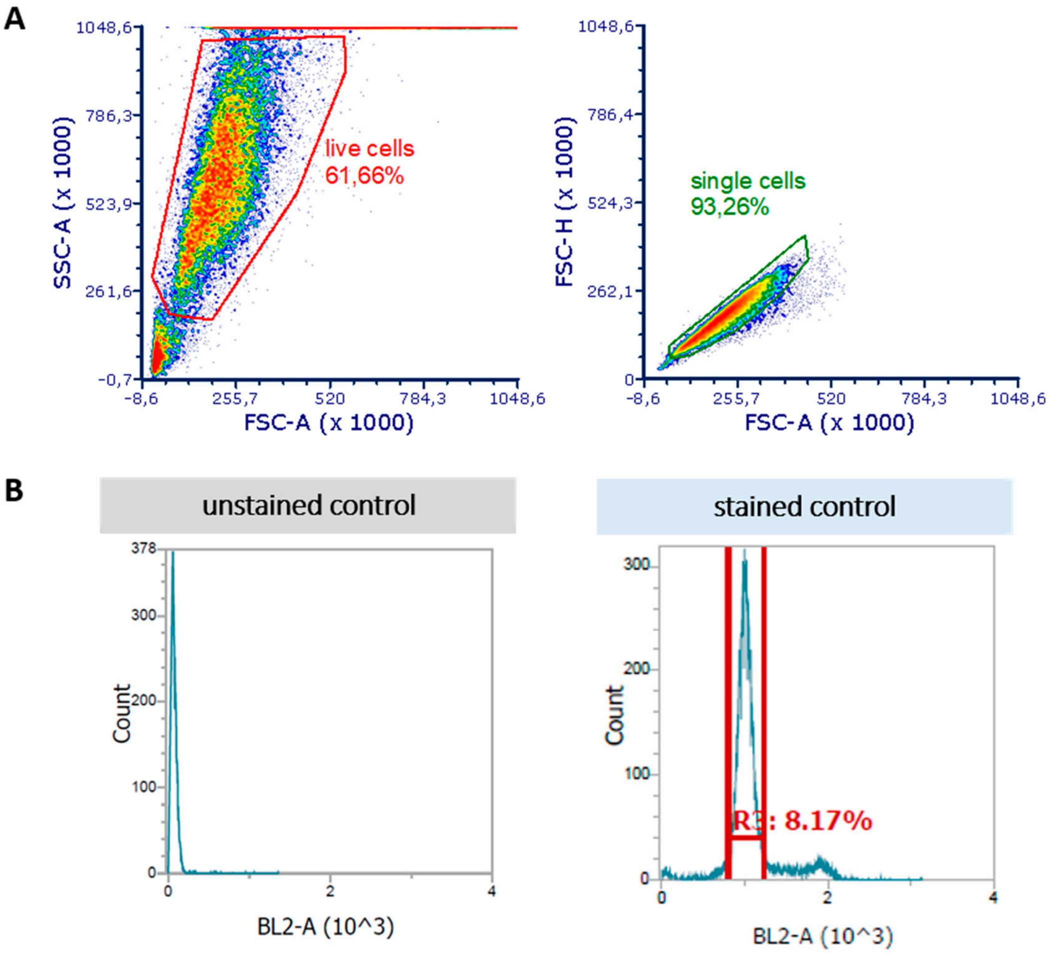

Supplementary Figure S1: Additional measurement information of cell cycle analysis (results shown in **Fig. 5**). Gating strategy (A). Additional histograms of unstained and stained control (propidium iodide: BL2-A) (B).

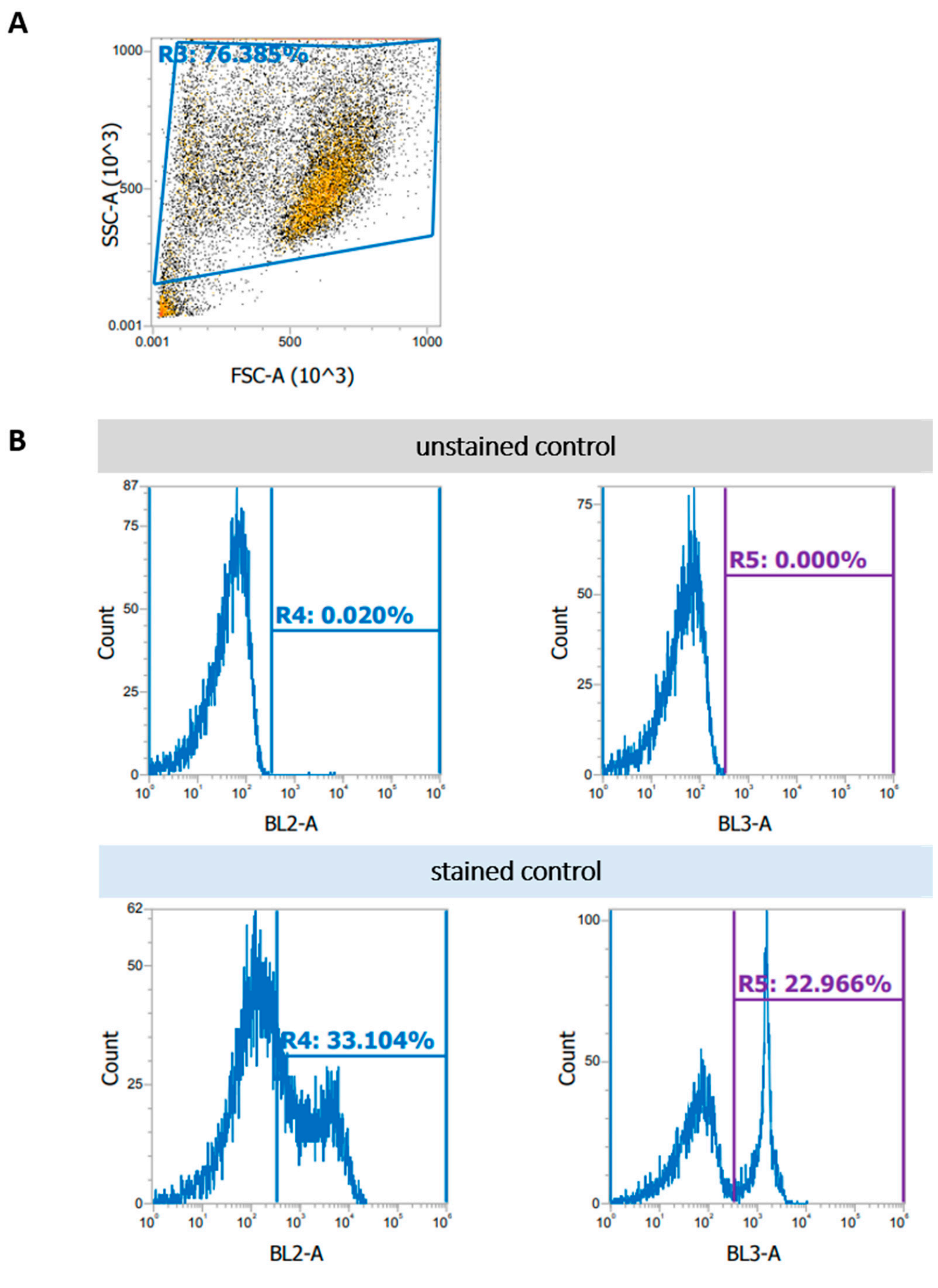

Supplementary Figure S2: Additional measurement information of apoptosis and necrosis analysis (results shown in **Figs. 3** and **9**). Gating strategy (A). Additional histograms of unstained and stained control (PE Annexin V: BL2-A; 7-AAD: BL3-A) (B).

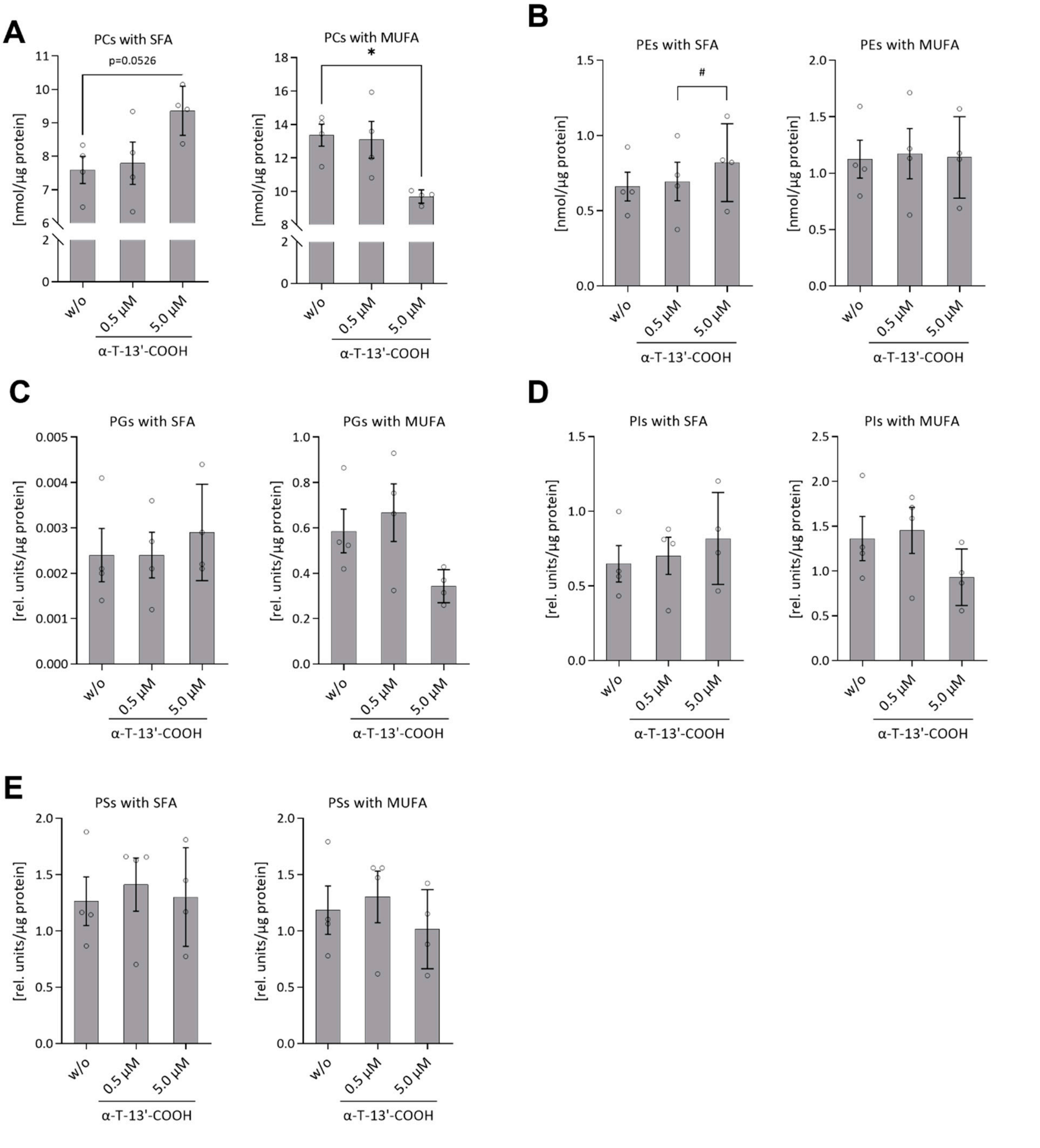

Supplementary Figure S3: Absolute amount of SFA- or MUFA-containing species of PC (A), PE (B), PG (C), PI (D) and PS (E). RAW264.7 cells were incubated with 0.5 or 5.0 µM α-T-13'-COOH for 24 h. The content of different species of TGs and phospholipids was then analyzed using UPLC-MS/MS. Experiments were performed in four independent biological replicates (points). Data are presented as means ± standard error of the mean (SEM). p-Values were calculated using repeated measurement one-way ANOVA with Tukey's post-hoc test. *, $p < 0.05$ (w/o *vs*. α-T-13'-COOH) or #, $p < 0.05$ (0.5 µM α-T-13'-COOH *vs.* 5.0 µM α-T-13'-COOH).

TGs			24 h
α-T-13'-COOH	w/o	0.5 µM	5.0 µM
18:1_16:1_18:2	1.03	0.98	0.31
18:0_18:2_18:2	1.54	1.59	0.50
18:1_16:1_22:6	0.33	0.25	0.11
18:0_16:1_20:3	0.52	0.61	0.19
18:1_16:1_16:1	3.02	2.82	1.12
18:0_18:1_18:1	9.40	9.14	4.04
16:0_18:0_18:2	3.11	3.20	1.53
16:0_16:1_22:5	0.19	0.18	0.10
16:0_18:1_18:1	30.21	29.60	15.70
16:0_16:1_18:1	14.81	14.96	8.48
16:0_16:1_18:2	0.98	1.13	0.57
18:0_18:0_18:1	1.78	1.86	1.13
16:0_16:1_20:3	0.47	0.48	0.42
16:0_18:0_18:1	5.65	6.45	5.27
16:0_16:0_18:2	0.79	0.95	0.95
16:0_16:0_22:5	0.12	0.15	0.16
16:0_16:0_18:1	8.74	8.63	11.71
16:0_16:0_16:1	9.99	8.88	13.50
16:0_16:0_22:6	0.21	0.21	0.28
16:0_18:0_18:0	0.75	0.82	1.85
16:0_16:0_20:4	0.21	0.21	0.53
16:0_16:1_16:1	0.33	0.37	0.95
16:0_16:0_18:0	1.98	2.21	6.92
16:0_16:0_16:0	3.63	4.23	22.32
18:0_18:0_18:0	0.18	0.10	1.38

0%
800%

PCs			24 h
α-T-13'-COOH	w/o	0.5 µM	5.0 µM
18:1_22:5	0.14	0.13	0.06
18:1_20:3	1.01	1.00	0.41
18:1_20:4	0.49	0.46	0.22
18:1_22:6	0.18	0.17	0.08
18:1_22:4	0.07	0.06	0.03
18:1_18:1	31.51	29.85	15.98
18:0_16:1	0.01	0.00	0.00
18:1_20:1	2.46	2.36	1.37
16:1_18:1	10.50	10.07	6.31
14:0_18:1	1.46	1.45	0.97
16:1_22:1	0.12	0.13	0.09
16:1_16:1	2.16	2.17	1.56
16:0_18:2	1.15	1.10	0.99
18:0_22:6	0.04	0.04	0.04
18:0_22:5	0.05	0.05	0.05
16:0_20:3	0.61	0.68	0.62
14:0_16:1	0.62	0.68	0.63
18:0_20:4	0.16	0.18	0.18
18:1_20:5	0.05	0.05	0.06
16:0_22:6	0.08	0.08	0.10
16:0_18:1	17.22	17.87	21.35
18:0_18:1	9.63	10.55	12.13
16:0_22:5	0.08	0.09	0.11
16:0_20:4	0.29	0.31	0.41
18:0_18:2	0.43	0.49	0.65
16:0_16:1	9.25	9.81	15.08
14:0_16:0	1.90	1.98	3.63
16:0_16:0	8.33	8.22	16.90

0%
>200%

PIs			24 h
α-T-13'-COOH	w/o	0.5 µM	5.0 µM
18:1_20:3	6.16	5.98	1.93
18:1_18:2	2.33	2.27	1.28
18:1_18:1	44.37	44.40	27.15
18:1_20:5	0.19	0.17	0.12
18:0_20:3	9.53	9.80	6.37
18:1_20:1	0.97	1.04	0.66
18:1_20:4	3.92	3.49	2.76
16:1_18:1	5.15	5.07	4.14
18:0_20:2	3.38	3.66	3.06
16:0_20:3	0.78	0.78	0.78
18:0_18:2	1.86	1.80	2.27
16:0_22:6	0.06	0.06	0.11
18:0_22:6	0.25	0.23	0.44
18:0_20:4	4.71	4.31	8.28
18:0_18:1	9.46	9.94	17.02
18:0_22:5	0.41	0.43	0.75
14:0_18:1	0.26	0.27	0.48
16:0_18:2	0.34	0.37	0.71
16:0_22:5	0.12	0.12	0.28
16:0_18:1	3.68	3.85	10.60
16:0_20:4	0.60	0.48	1.83
18:0_16:1	0.76	0.78	2.96
16:0_16:1	0.56	0.54	2.17
16:0_16:0	0.15	0.17	3.88

0%
>500%

A	B	C
D	E	F

PSs			24 h
α-T-13'-COOH	w/o	0.5 µM	5.0 µM
16:1_18:1	2.02	1.86	1.00
18:1_18:1	14.07	13.33	7.87
16:1_22:1	2.51	2.34	1.62
16:0_16:0	0.73	0.61	0.49
18:1_20:1	1.46	1.47	1.09
18:0_20:3	4.16	4.40	3.92
18:0_18:1	36.91	37.88	35.71
18:0_16:1	7.39	7.23	7.17
18:0_18:2	2.35	2.40	2.52
18:0_22:5	6.26	6.27	7.31
16:0_16:1	2.19	2.15	2.63
18:0_20:4	3.27	3.25	4.01
18:0_22:6	7.28	7.34	9.28
16:0_18:1	7.95	7.97	11.76
16:0_20:4	0.45	0.45	0.99
16:0_22:5	0.57	0.57	1.42
16:0_22:6	0.45	0.46	1.22

0%
300%

PGs			24 h
α-T-13'-COOH	w/o	0.5 µM	5.0 µM
18:1_18:1	86.12	86.24	83.77
18:1_22:5	0.94	0.94	0.93
18:1_22:6	1.85	1.93	1.99
16:1_18:1	9.74	9.61	11.30
18:1_20:4	0.95	0.92	1.18
16:0_18:2	0.40	0.36	0.83

0%
>200%

PEs			24 h
α-T-13'-COOH	w/o	0.5 µM	5.0 µM
18:1_22:4	0.26	0.25	0.17
18:1_18:1	29.35	29.16	20.73
18:1_22:5	0.42	0.40	0.30
18:1_18:2	0.93	0.92	0.68
18:1_22:6	0.34	0.33	0.25
18:1_20:4	2.09	2.10	1.61
16:1_18:1	4.14	4.00	3.25
18:0_18:1	40.83	40.71	42.69
18:0_18:2	1.78	1.84	1.96
18:0_22:6	0.78	0.82	0.97
18:0_16:1	2.86	2.91	3.62
18:0_22:5	1.56	1.62	1.98
18:0_20:4	4.26	4.30	5.89
16:0_18:1	6.52	6.57	9.10
16:0_16:1	2.08	2.20	3.38
16:0_22:5	0.18	0.19	0.30
16:0_18:2	0.34	0.36	0.61
16:0_20:4	0.78	0.82	1.50
16:0_16:0	0.35	0.33	0.69
16:0_22:6	0.14	0.16	0.30

0%
>200%

Supplementary Figure S4: Impact of α-T-13’-COOH on the fatty acid composition of TGs and phospholipids. RAW264.7 cells were incubated with 0.5 or 5.0 µM α-T-13‘-COOH for 24 h. The content of different species of TGs and phospholipids was then analyzed using UPLC-MS/MS. Heatmap showing the cellular proportions of indicated lipid species relative to the total lipid subclass (100%) (A: TG; B: PC; C: PI; D: PS; E: PG; F: PE). Experiments were performed in four independent biological replicates and the data are presented as mean value. Color indicates the fold change in the cellular proportions compared to the vehicle control. Red indicates elevated (> 100%) and blue reduced cellular proportions (< 100%) as compared to vehicle control.

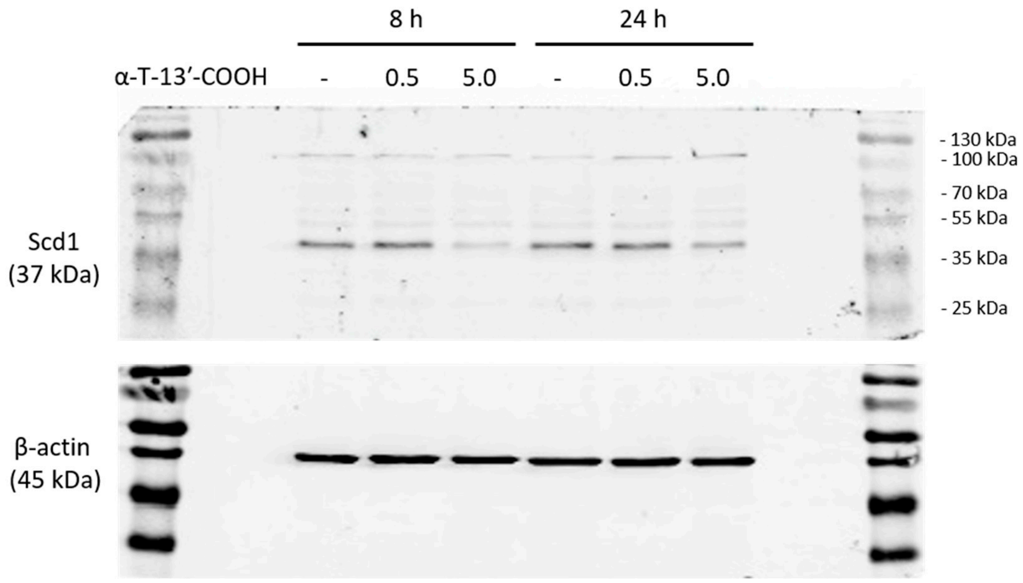

Supplementary Figure S5: Unprocessed Western Blot images of Scd1 protein (whole cell lysate) shown in **Fig. 8**.

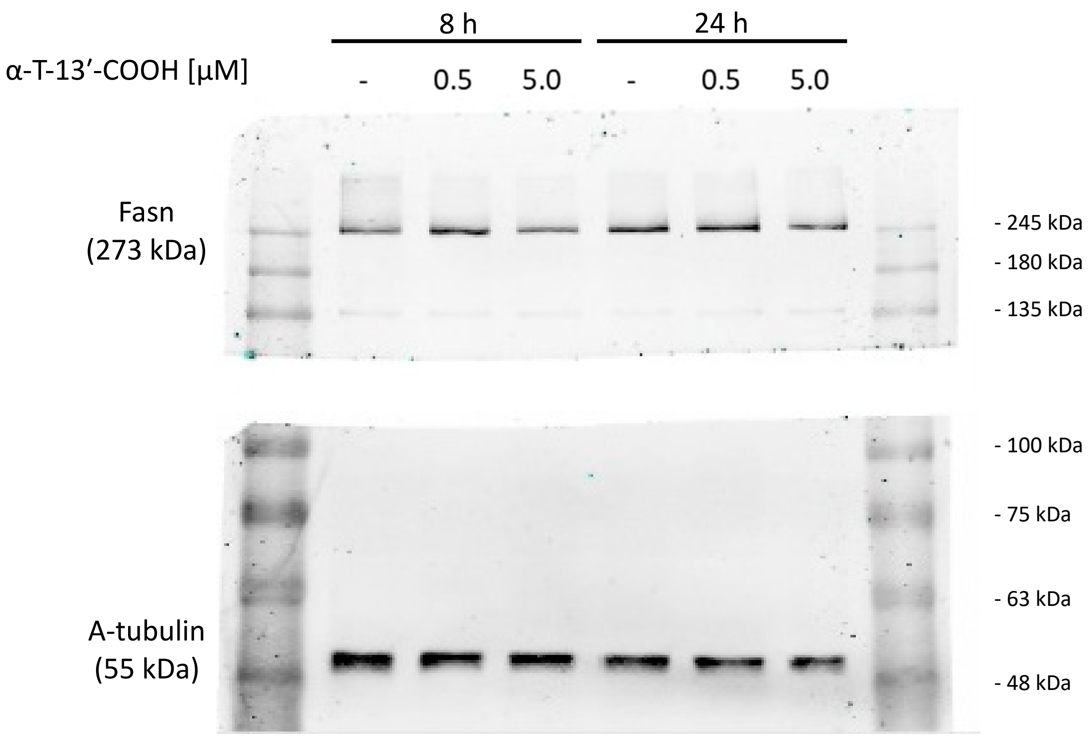

Supplementary Figure S6: Unprocessed Western Blot images of Fasn protein (whole cell lysate) shown in **Fig. 8**.

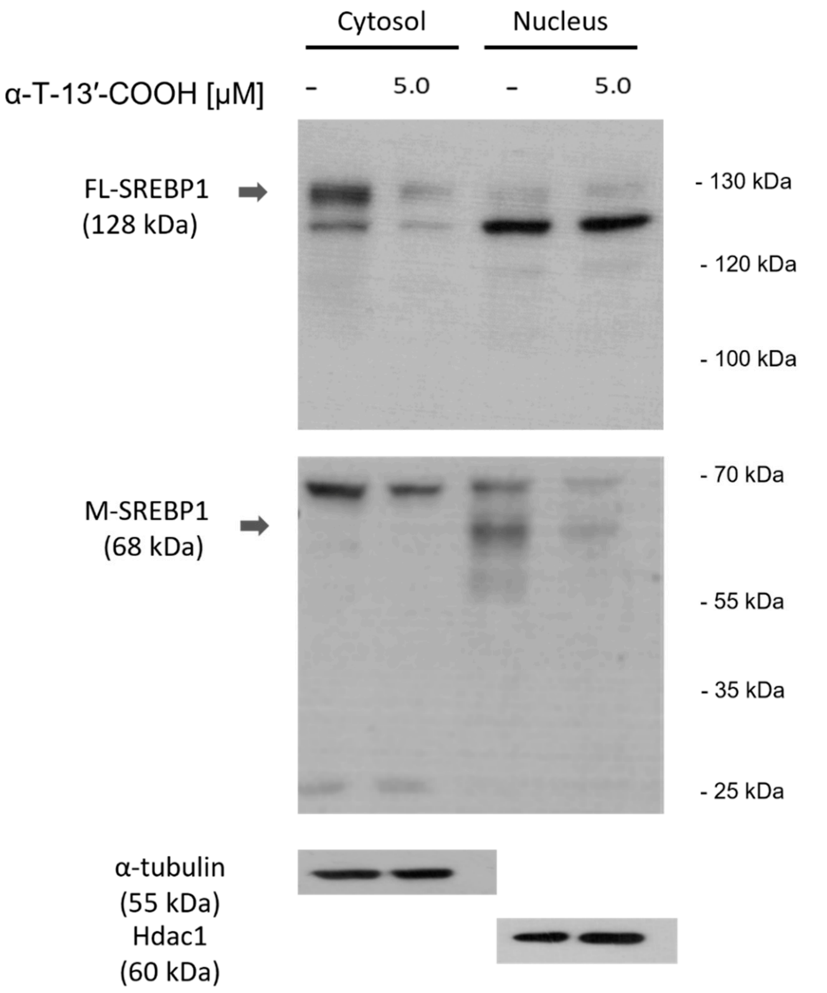

Supplementary Figure S7: Unprocessed Western Blot images of Srebp1 protein (cytosol and nucleus) shown in **Fig. 10**. The membrane was separated (25-70 kDa and 100-130 kDa) after blotting to use different incubation conditions as described in method section.

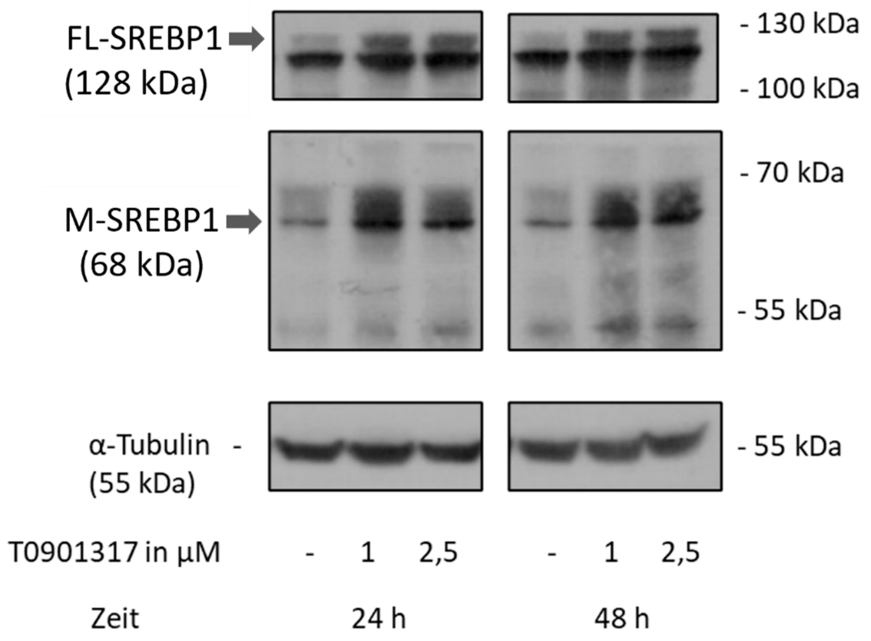

Supplementary Figure S8: Validation of the specificity of the mouse anti-Srebp1 specificity antibody in RAW264.7 macrophages. Cells were treated with vehicle (DMSO) or the LXR agonist T0901317 for 24 or 48 h. Protein expression of Srebp1 (full-length and mature forms) was analyzed by Western blot in whole cell lysates. The bands marked with arrows increased in intensity upon treatment with T0901317 and are designated as full-length and mature forms of Srebp1, respectively.
